# Compound drivers of Antarctic sea ice loss and Southern Ocean destratification

**DOI:** 10.1126/sciadv.aeb0166

**Published:** 2026-05-08

**Authors:** Aditya Narayanan, Holly Ayres, Matthew H. England, F. Alexander Haumann, Matthew R. Mazloff, Alessandro Silvano, Theo Spira, Shenjie Zhou, Alberto C. Naveira Garabato

**Affiliations:** ^1^ Ocean and Earth Science, University of Southampton, Southampton, UK.; ^2^ Center for Marine Science and Innovation, and ARC Australian Centre for Excellence in Antarctic Science, University of New South Wales, Sydney, Australia.; ^3^ National Oceanography Centre, Southampton, UK.; ^4^ Alfred Wegener Institute, Helmholtz Centre for Polar and Marine Research, Bremerhaven, Germany.; ^5^ Ludwig-Maximilians-Universität München, Munich, Germany.; ^6^ Scripps Institution of Oceanography, University of California San Diego, La Jolla, CA 92093, USA.; ^7^ Department of Marine Science, University of Gothenburg, Gothenburg, Sweden.; ^8^ British Antarctic Survey, Cambridge, UK.

## Abstract

Antarctic sea ice extent began declining in 2015, reaching its minimum in the post-1970s observational era in 2023. To diagnose the drivers of this decline, we analyze an observationally constrained sea ice–ocean model spanning 2013–2023 and identify three distinct phases of sea ice retreat. First, intensifying westerlies preconditioned the Southern Ocean via increased upwelling of warm, saline circumpolar deep water (CDW). Second, strong winds in 2015–2016 enhanced the mixing of CDW into the upper ocean, thereby initiating sea ice loss, particularly in East Antarctica. Third, sustained mixing of CDW into the surface layer, combined with reduced equatorward sea ice–derived freshwater export, maintained an unprecedentedly low sea ice state. East Antarctic sea ice loss was primarily subsurface driven via enhanced upward CDW flux, whereas West Antarctic sea ice loss was also forced by longwave radiative flux anomalies. Our findings suggest that persistent upwelling-favorable conditions under anthropogenic forcing may push the Southern Ocean into a prolonged low sea ice state.

## Teaser

Recent sea ice loss was initiated by ocean upwelling in East Antarctica and by radiative flux anomalies in West Antarctica.

## INTRODUCTION

Antarctic sea ice is an important component of the global climate system, modulating the albedo of the Southern Ocean (*1*), the upper and lower branches of the meridional overturning circulation (*2*), the water mass transformations therein (*3*), oceanic heat and carbon uptake (*4*), ocean heat content (*5*), and biological productivity (*6*). Antarctic sea ice exhibited a slight positive trend from 1979 to 2015 (*7*–*9*), with large regional variations. The expansion of sea ice over this period was most likely associated with an increased wind-driven northward transport of sea ice (*10*) and a resulting surface freshening due to the export of sea ice–derived freshwater from the high-latitude Southern Ocean (*11*). However, since 2015, the observed sea ice area has experienced persistent negative anomalies, with the lowest wintertime and summertime sea ice extents (SIEs) measured in 2023 (*12*). The negative SIE anomalies were associated with heightened temperatures in the upper 100 m of the water column (*13*) and elevated surface salinity (*14*). This rapid change in Southern Ocean sea ice from record-high to record-low anomalies is one of the largest present-day climatic shifts in the Earth system and has the potential to accelerate planetary warming (*1*) and to disrupt the conventional pathways for heat and carbon sequestration in the Southern Ocean (*15*). In addition, sea ice loss can adversely affect the ecosystem (*16*) and may also affect the stability of ice shelves that buttress glaciers vulnerable to ocean warming (*17*).

Several hypotheses have been proposed to explain the roles of the ocean and atmosphere in modulating the recent reduction of Antarctic SIE. Revisiting these hypotheses by categorizing them based on the timescales of the proposed mechanisms can provide valuable insight. On synoptic timescales, wind variations can immediately influence the ocean’s Ekman advection. For example, during the summers of 2016/17 and 2019/20, a sudden weakening of the westerlies resulted in reduced northward Ekman advection of relatively cool and fresh surface waters, leading to surface warming and salinification in the offshore subpolar Southern Ocean (*18*). This warming may have contributed to the summertime reduction in sea ice area and subsequent delayed sea ice growth. On seasonal timescales, intense polar cyclones were likely linked to the formation of open-ocean polynyas in the Weddell Sea in 2016 and 2017 (*19*–*21*). In addition, a positive Zonal Wave-3 (ZW3) pattern (*22*) during 2016 was associated with stronger poleward transport of warm subtropical air masses, enhancing cloud cover and downward longwave radiative fluxes over the sea ice field (*19*, *23*). The spatiotemporal trajectory of the ZW3 pattern in 2016 influenced sea ice concentration and drift, contributing to reduced SIE in the Weddell Sea, the Amundsen and Bellingshausen Seas, and the western Ross Sea (*23*–*26*). Warm northerly air flow was linked to a deepening of the Amundsen Sea Low (ASL) in 2016 and 2019 (*21*, *27*).

On seasonal to interdecadal timescales, the Southern Annular Mode (SAM) is the dominant mode of climate variability over the high-latitude regions of the Southern Ocean (*28*). A positive phase of the SAM is associated with a poleward shift and increased intensity of the westerlies, which regulate the rate of Ekman advection. An intensified SAM is thus expected to exert two opposing effects on upper-ocean stratification: surface freshening due to enhanced equatorward export of polar waters and sea ice (*11*, *29*) and surface salinification further south due to increased Ekman pumping (*30*). The SAM has trended positive since the 1970s (*28*), associated with an enhancement in the cyclonicity of winds over the subpolar Southern Ocean that has increased Ekman pumping of warm deep waters from the subsurface (*24*, *31*, *32*). Further, the SAM has exhibited increasing zonal asymmetry due to a deepening of the ASL and an intensification of the ZW3 pattern (*33*), which enhanced the poleward flow of warm, humid subtropical air masses within specific sectors of the Southern Ocean. Preindustrial runs of climate models show that the SAM-ZW3 interaction affects the regional variability of sea ice (*34*).

Future sea ice evolution is likely to be governed by a balance between competing mechanisms. For example, the heat content in the ocean and atmosphere is expected to continue to increase under anthropogenic forcing, which would inhibit sea ice growth. In contrast, enhanced surface freshening (*35*) due to increased precipitation (*36*) and glacial meltwater input (*37*) is expected to stratify the upper ocean and cause a slowdown in the abyssal overturning cell (*37*, *38*), which would promote sea ice growth by inhibiting the vertical mixing of heat. However, in the present-day Southern Ocean, a somewhat unexpected trend of upper-ocean salinification is occurring (*14*). Such salinification weakens stratification (*14*) and reverses the stabilizing influence of freshwater, potentially enabling the mixing of heat and salt from the subsurface circumpolar deep water (CDW) layer.

The possible role of increased westerly winds in determining the fate of Antarctic sea ice was highlighted by a hypothesis based on idealized model studies. In this view, poleward intensifying westerlies would elicit a two-timescale response from the ocean (hereafter referred to as the two-timescale hypothesis) (*39*). The immediate response would involve enhanced northward Ekman transport of cooler and fresher waters from the high-latitude Southern Ocean, inducing surface cooling and enhanced sea ice cover. The delayed response would be associated with enhanced upward Ekman pumping of warm and saline CDW, bringing about a warmer and saltier upper ocean with reduced sea ice cover. However, the observed ocean response is more complex, influenced also by surface freshening (*5*) and mesoscale eddy activity (*40*, *41*) that may counteract the wind-driven changes to stratification and overturning. Recent, more realistic simulations further suggest that reduced SIE can result from upwelling-favorable conditions arising either from natural Southern Ocean variability (*42*) or from historically forced conditions (*43*). Overall, the observed pattern of a gradual increase followed by an abrupt reduction in Antarctic sea ice cover after 2015 qualitatively aligns with expectations from the two-timescale hypothesis (*13*), although substantial differences remain.

While many potentially important processes have been proposed, the mechanisms governing the recent climatic evolution of Antarctic sea ice remain uncertain and are the focus of vigorous scientific discourse. Climate models generally struggle to represent the observed variability and often simulate physically implausible scenarios (*44*). Here, we use an eddy-permitting, data-assimilative sea ice–ocean state estimate—the Biogeochemical Southern Ocean state estimate (SOSE) (*45*)—to elucidate the drivers of Antarctic sea ice changes between 2013 and 2023, a period encompassing the point of abrupt reduction in ice cover from record highs to record lows (*46*). By constructing budgets of sea ice volume (SIV) and conserved upper-ocean properties (such as heat and salt), we are able to identify the key factors in sea ice loss and assess its forcing mechanisms and underpinning sequence of causal events.

Our analysis shows that the recent Antarctic sea ice loss was the compound outcome of three driving phases. First, before mid-2015, SIE increased in association with cool and fresh anomalies in the upper ocean, a pattern consistent with enhanced equatorward export of sea ice–derived polar freshwater that stabilized the surface. Second, after mid-2015, heat and salt accumulated in the upper ocean, initially as a result of the shoaling of warm and saline CDW. This response, qualitatively consistent with the two-timescale hypothesis, was facilitated by upwelling-favorable winds and by enhanced vertical mixing of heat and salt, itself driven by intensified westerly winds. Then, in a third phase, the preceding sea ice changes altered surface freshwater fluxes, which became increasingly important in sustaining elevated salinity and weakened stratification in the upper ocean after 2018—thus promoting the persistence of a reduced Antarctic sea ice state.

Last, we reveal substantial differences in the sea ice evolution and its driving mechanisms between East versus West Antarctica. This zonal asymmetry stems from corresponding contrasts in wind forcing, highlighting the spatial complexity of Southern Ocean coupled atmosphere-ice-ocean dynamics. By identifying the dominant mechanisms within each region, we provide an integrated picture of circumpolar Antarctic sea ice changes.

## RESULTS

### Overview of Southern Ocean hydrographic evolution

**Fig. 1.**
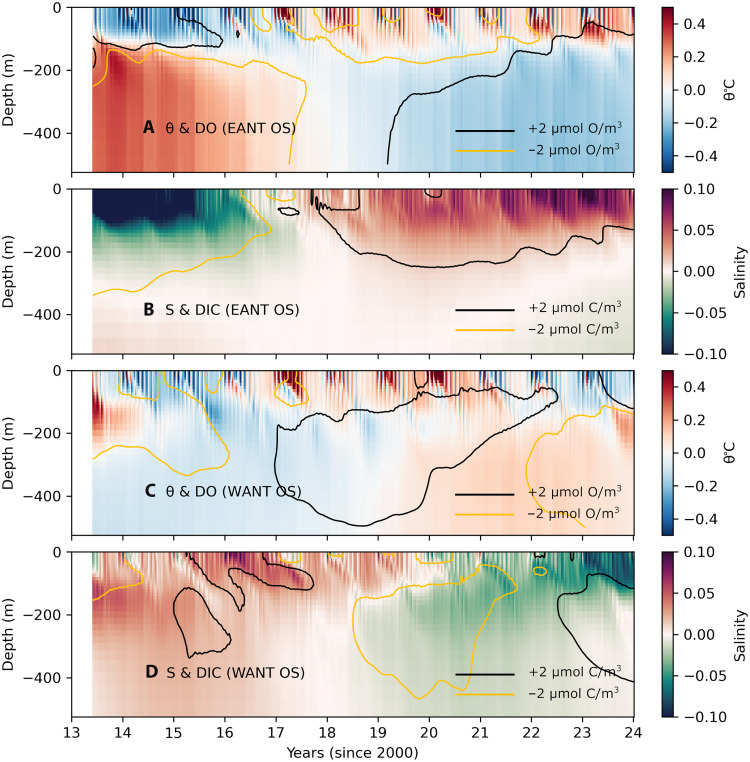
SOSE hydrography. SOSE potential temperature and salinity anomalies in (**A** and **B**) East Antarctica’s off-shelf (EANT OS) and (**C** and **D**) West Antarctica’s off-shelf (WANT OS) regions. Contours in the temperature panels represent DO anomalies (±2 μmol O/m^3^), while contours in the salinity panels represent DIC anomalies (±2 μmol C/m^3^). Negative anomalies are indicated by yellow contours, and positive anomalies are shown with black contours.

In the subpolar Southern Ocean, CDW lies just below the pycnocline in weakly stratified waters during winter (figs. S1 and S2) (*47*). Relative to surface waters, CDW is characterized as warmer and saltier with lower concentrations of dissolved oxygen (DO) and higher concentrations of dissolved inorganic carbon (DIC) (*48*). The off-shelf regions of East Antarctica (E Ant; between 60°W and 150°E; see Methods) exhibit a subsurface (below 100 m) warm anomaly and an upper-ocean cool and fresh anomaly during 2013–2016. Subsequently, the upper ocean (upper 100 m) becomes warmer and saltier, while beyond 2018, the subsurface ocean cools and becomes slightly fresher between depths of 200 and 500 m (Fig. 1, A and B). The surface warming and salinification are accompanied by a depletion in DO (yellow contours in Fig. 1A) and an increase in DIC (black contours in Fig. 1B) in the upper ocean.

In West Antarctica (W Ant; between 150°E and 60°W; see Methods), the upper ocean exhibits a cool anomaly from 2013 to 2015, a warm anomaly from 2016 to 2020, and another cool anomaly from 2021 to 2023 (Fig. 1C). The upper ocean shows DO depletion from 2013 to 2019 (yellow contours; Fig. 1C). The subsurface ocean displays DO enrichment between 2017 and 2021 (black contours; Fig. 1C). The upper ocean exhibits a salty anomaly from 2013 to 2019 (Fig. 1D), with DIC enrichment observed from 2015 to 2019 (black contours; Fig. 1D). After 2020, the upper ocean transitions to a fresh anomaly. The subsurface ocean shifts from cooler and saltier anomalies during 2013–2018 to warmer and fresher anomalies after 2018. This is in contrast with East Antarctica, where the subsurface transitions from warm and salty to cool and fresh anomalies.

To provide additional context to these hydrographic changes, we include the actual temperature and salinity fields in fig. S2. These show that East Antarctica generally has a more saline upper ocean in winter than West Antarctica. The internal pycnocline varies seasonally in both regions, lying at shallower depths in winter and deepening in summer. East Antarctica also exhibits a shallower winter pycnocline (50 m) compared with West Antarctica (80 m).

In summary, beginning in mid-2015, the upper ocean off East Antarctica undergoes a clear transition from cooler, fresher, DO-enriched, and DIC-depleted waters to a warmer, saltier, DO-depleted, and DIC-enriched state. These anomalous properties are characteristic of CDW (*48*), thereby indicating an increased presence of CDW in the near-surface ocean, consistent with observations (*32*). We will pin down the mechanisms involved by carrying out detailed analyses of heat and salt budgets in the following sections. In contrast, the hydrographic evolution off West Antarctica appears more complex. While there is a clear transition from a saltier upper ocean (2013–2019) to a fresher upper ocean (2020–2023), the other parameters exhibit more convoluted changes.

The upper-ocean warming and salinification associated with sea ice loss seen in SOSE are consistent with studies based on in situ hydrographic profiles (*13*) and remotely sensed sea surface salinity (*14*). Here, we show that there is a zonal asymmetry in the evolution of upper-ocean hydrographic properties. Further, the mechanisms behind these changes have not yet been explored. In the following sections, we will consider budgets of SIV, temperature, and salinity to gain greater clarity on the dynamics governing the variability in Antarctic sea ice.

### Antarctic SIE and volume anomalies

**Fig. 2.**
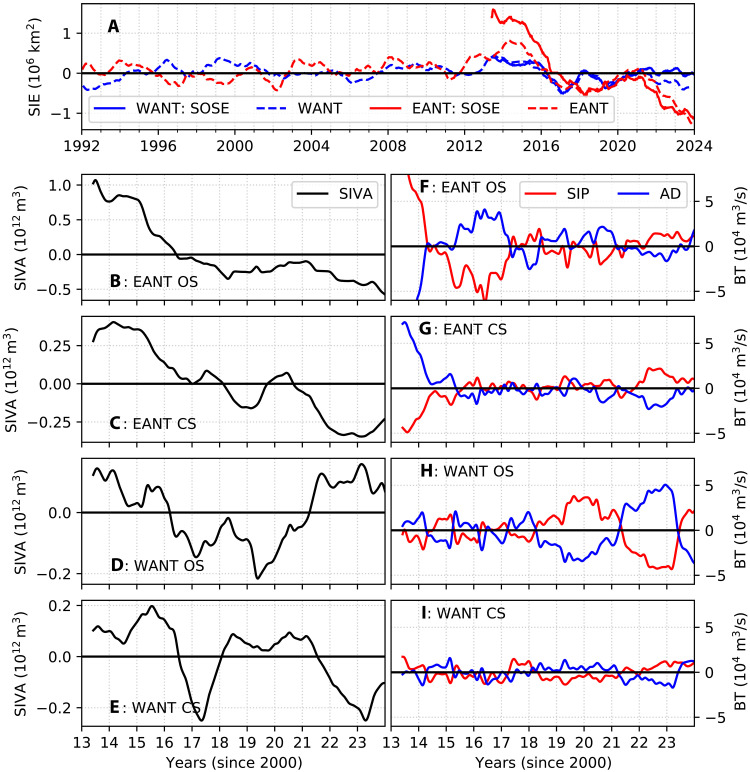
Sea ice budgets. (**A**) SIE anomalies computed from satellite observations (broken line) and SOSE (solid lines) for E Ant (EANT; red lines) and W Ant (WANT; blue lines). (**B** to **E**) Sea ice volume anomalies (SIVA; 10^12^ m^3^), spatially summed over the continental shelf (CS) and off-shelf (OS) regions of E Ant and W Ant. Note that the *Y* axis scale differs for each region. (**F** to **I**) Sea ice volume budget terms (BT) for the corresponding regions are shown on the right-hand column. The terms, represented as anomalies, are the sea ice production (SIP; red line; computed as a residual of Eq. 2) and the advection and divergence (AD; blue line) of SIV. Time-integrated budget terms are shown in fig. S4.

Satellite observations reveal that negative SIE anomalies persisted beyond 2016 in both East Antarctica and West Antarctica (Fig. 2A). Earlier observations indicate relatively stable sea ice with a zonal see-saw pattern in sea ice anomalies between West Antarctica (Ross, Amundsen, and Bellingshausen Seas; see Methods) and East Antarctica (all regions outside West Antarctica), characterized by oppositely signed anomalies across these regions before 2008. The time tendency of SIE anomalies in SOSE aligns with satellite observations, although their magnitude exhibits some positive bias with respect to observations from 2013 to 2016 in East Antarctica and from 2022 to 2023 in West Antarctica. In addition, the temporal tendency of anomalies in SOSE generally matches that observed in satellite data. SIE anomalies largely reflect anomalies at the equatorward margin of the sea ice pack, but sea ice thickness anomalies in SOSE show that sea ice was reduced well within the pack as well (fig. S3). Both East Antarctica and West Antarctica display negative SIV anomalies after 2016, with the off-shelf West Antarctic sea ice showing a recovery beyond 2021 (Fig. 2, B to E). This recovery period coincides with a positive bias in SOSE SIE. We discuss the reasons for this in later sections.

Budgets reveal that the loss in SIV in the off-shelf (bathymetry deeper than 3000 m) East Antarctic region was driven by a drop in net thermodynamic sea ice production (SIP; i.e., growth minus melt; Fig. 2F and fig. S4F). This signal is consistent with earlier studies that suggested that sea ice loss beyond 2015 was largely thermodynamically forced, rather than mechanically driven by advection or divergence (*49*). However, because of uncertainties in sea ice thickness measurements, earlier studies could not quantify the volume loss now made evident by these results. While sea ice advection and divergence (AD) partially offset the decline in SIP, this was insufficient for full compensation, resulting in a negative anomaly in SIV in East Antarctica (Fig. 2B). The additional contribution to SIV from the AD term arises from enhanced SIP on the continental shelves of East Antarctica during 2015–2016, 2019–2020, and 2022–2023 (Fig. 2G). This enhancement is likely due to increased sea ice export from the continental shelves, facilitated by reduced SIP in the off-shelf regions. A negative SIV anomaly in both the continental shelf and off-shelf regions of East Antarctica was seen after 2020 (Fig. 2, F and G).

Sea ice volume loss in the off-shelf West Antarctic region is not as pronounced and does not last for the entire period beyond 2016 (Fig. 2; West Antarctica’s off-shelf and West Antarctica’s continental shelf). Low SIV, seen between 2016 and 2020, was driven by a combination of factors involving both low SIP (during 2013, 2015, 2017, and in 2021–2022) and low AD (evident during 2018–2020; Fig. 2H and fig. S4H). Budgets of SIV in SOSE reveal a zonal asymmetry in sea ice evolution, with East Antarctica showing relatively a more persistent negative anomaly in SIV beyond 2016 (Fig. 2; East Antarctica’s off-shelf and East Antarctica’s continental shelf). The evolution of SIV anomalies are consistent with the observed SIE anomalies, with the losses being more prominent in East Antarctica.

The SIV budgets highlight the critical role of thermodynamics in reducing sea ice in East Antarctica, suggesting that the mechanisms driving sea ice loss involve heat transfer to the ice. Heat can be sourced from the atmosphere above or from the warm CDW, typically found below the pycnocline. The sea ice loss was associated with a warming and salinification of the upper ocean along with an accumulation of DIC and a depletion of DO. These signatures are consistent with a greater near-surface presence of CDW (*50*), but they could also arise from enhanced atmospheric heat input. Distinguishing between these possibilities requires a detailed analysis of the processes controlling the supply of heat to the upper ocean, presented in the following section.

In the large subpolar gyres of the Weddell and Ross Seas, the pycnocline (and underlying CDW layer) shoals to depths as shallow as 50 to 100 m (*32*, *47*). However, this heat remains trapped below the pycnocline unless stratification is sufficiently weakened to facilitate mixing of CDW into the surface mixed layer (*51*, *52*). In cold polar waters, stratification is primarily controlled by salinity (*53*). To investigate the processes that lead to the weakening of stratification and the upward transfer of heat into the mixed layer, we analyze the upper-ocean temperature and salinity budgets in the next two sections.

### Upper-ocean warming in the Southern Ocean

Shortwave and longwave fluxes are primarily influenced by two factors: (i) sea ice, which alters surface albedo, and (ii) cloud cover, which reduces the transmission of shortwave radiation through the atmosphere but enhances downward longwave radiation. The subpolar Southern Ocean is generally characterized by a net heat loss via longwave radiation. Therefore, positive anomalies in downward longwave flux represent a reduction in this heat loss, effectively contributing to ocean warming.

**Fig. 3.**
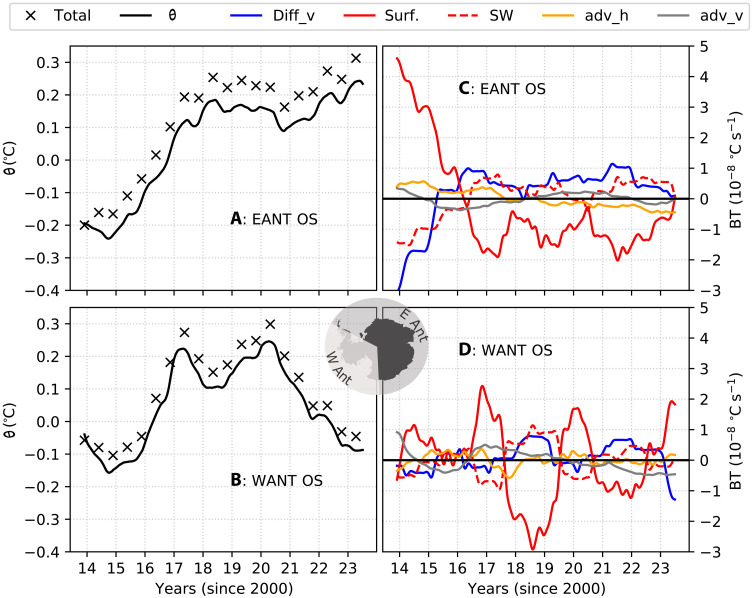
SOSE temperature budgets. (**A** and **B**) Potential temperature (θ; black line with cross markers), vertically averaged in the upper 100 m of the water column and spatially averaged over the regions labeled within the panels. A 12-month rolling mean was applied to remove the seasonality. The time-integrated sum of the budget terms is shown by cross markers. (**C** and **D**) θ Budget terms are presented here as anomalies relative to their monthly means. Terms shown are the vertical advection (adv_v; gray lines), horizontal advection (adv_h; orange lines), vertical diffusion (Diff_v; blue lines), surface fluxes (surf; red lines), and shortwave (SW) fluxes (broken red lines). Individual time-integrated budget terms are shown in fig. S6.

Applying this framework to the off-shelf regions of East Antarctica, upper-ocean warming is evident from 2015 to 2018 (Fig. 3A). From late 2013 to mid-2015, surface fluxes (“surf”; excludes shortwave fluxes) show positive anomalies (a warming tendency) during periods of expanded sea ice cover. This is consistent with the insulating effect of sea ice that suppresses heat loss to the atmosphere via longwave and sensible heat fluxes (years 2013–2014; red line in Fig. 3C). From early 2016, surface fluxes exhibit a prominent and sustained negative anomaly, reflecting greater heat loss to the atmosphere due to reduced sea ice cover (surf; red line in Fig. 3C) (*12*).

Despite this shift, a key driver of upper-ocean warming beginning in mid-2015 is the vertical mixing term (Diff_v), which transitions from a negative to a sustained positive anomaly through 2023 (blue line in Fig. 3C). In contrast, shortwave fluxes (red dashed line in Fig. 3C) only shift to a positive anomaly in mid-2016, following the onset of sea ice loss in mid-2015. This timing suggests that shortwave fluxes did not initiate the sea ice decline but rather contributed to its subsequent intensification. Although surface fluxes warmed the ocean from late 2013 to early 2015, this warming was offset by cooling due to vertical mixing, resulting in little net temperature change. Starting in mid-2015, however, temperatures begin to rise (Fig. 3A), initially driven by the intensification of vertical mixing.

The off-shelf regions of West Antarctica also experience upper-ocean warming, beginning in 2015 and peaking between 2017 and 2020, followed by a period of cooling (Fig. 3B). However, the mechanisms driving this warming differ notably from those in East Antarctica. In East Antarctica, vertical mixing plays an early and sustained role, whereas in West Antarctica, vertical mixing contributes to warming only during specific years (2018, 2021, and 2022). Further, in contrast to East Antarctica, shortwave fluxes in West Antarctica exhibit negative anomalies even during periods of anomalously reduced sea ice cover, such as the summers of 2016/2017 and 2019/2020. Concurrently, the remaining surface flux components show positive anomalies. This pattern suggests warming due to reduced heat loss via longwave radiation in West Antarctica, in contrast to East Antarctica, where reduced sea ice consistently coincides with increased shortwave flux and negative anomalies in the other surface fluxes.

**Fig. 4.**
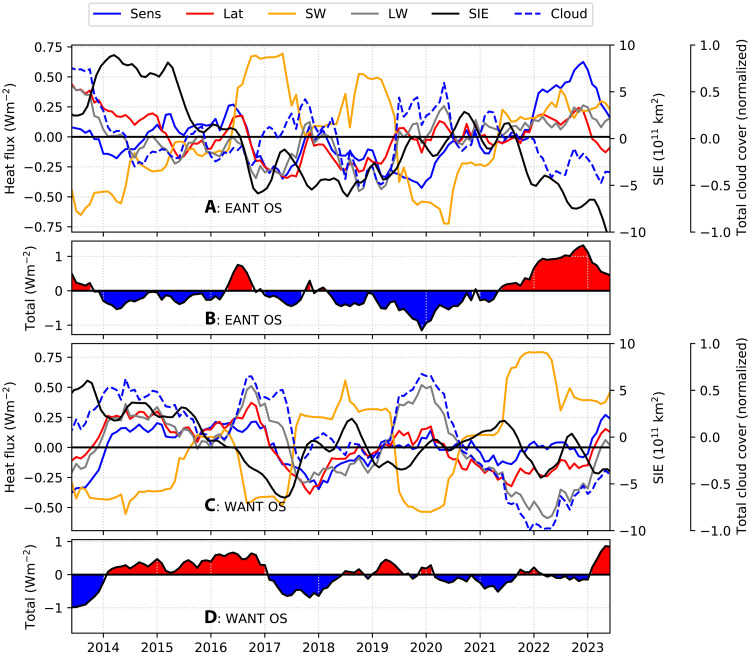
Surface heat flux anomalies from ERA5 over the off-shelf regions of Antarctica. (**A**) and (**B**) correspond to East Antarctica (EANT), while (**C**) and (**D**) correspond to West Antarctica (W ANT). For each region, the first panel [(A) and (C)] shows anomalies in individual flux components—shortwave (SW; orange), latent (Lat; red), sensible (Sens; blue), and longwave (LW; gray)—along with SIE anomaly (black, right-hand *Y* axis) and total cloud cover anomaly (Cloud; broken blue, normalized). The second panel for each region [(B) and (D)] shows the net surface heat flux anomaly. All anomalies are relative to monthly climatologies, and for the purpose of visualization, time series are smoothed with a 12-month rolling mean to remove seasonal variability.

To examine the components of surface heat fluxes in greater detail, we analyzed the European Centre for Medium-Range Weather Forecast version 5 (ERA5) over the off-shelf regions of East and West Antarctica. The results (Fig. 4) reveal patterns consistent with those observed in SOSE (Fig. 3). In East Antarctica, periods of enhanced SIE generally correspond to negative anomalies in shortwave fluxes: During periods of high SIE (positive anomalies), the time-mean anomalies are −0.3 W/m^2^ for shortwave and 0.1 W/m^2^ for longwave fluxes. Conversely, during periods of reduced SIE (negative anomalies), shortwave fluxes generally exhibit positive (warming) anomalies, while longwave fluxes usually display negative anomalies, with corresponding means of 0.19 and −0.06 W/m^2^ (Fig. 4A).

In West Antarctica, however, reduced SIE is not always associated with positive shortwave flux anomalies. During low-SIE periods such as the summers of 2016/2017 and 2019/2020, shortwave fluxes exhibit negative anomalies (cooling tendency), while longwave fluxes display positive anomalies (Fig. 4C). The time-mean anomalies of shortwave and longwave fluxes in these summers are −0.46 and 0.14 W/m^2^, respectively. These signals are linked to enhanced cloud cover over the region (broken blue line; Fig. 4C) and align with previous studies (*22*, *33*) that associate intensified cloudiness with the poleward advection of warm, humid subtropical air driven by a strengthened ZW3 pattern.

In East Antarctica, the total heat flux shows a brief positive anomaly in early to mid-2013, in mid-2016, and again from mid-2021 through 2023 (Fig. 4B). In contrast, in West Antarctica, the total heat flux exhibits warm anomalies early on and for a more sustained period from 2014 to late 2016, and once again from mid-2018 to early 2020, and in 2023 (Fig. 4D).

A warming anomaly is also evident in the net surface fluxes in 2023 (Fig. 4D), initiating renewed upper-ocean warming in West Antarctica (Fig. 3D). In the earlier sea ice budget analysis, we noted a divergence between SOSE and observations in 2022 and 2023, with SOSE failing to capture the observed sea ice decline. However, the heat budget indicates that SOSE does simulate renewed upper-ocean warming during this period—suggesting that sea ice loss may eventually follow. This points to a possible lag in SOSE’s sea ice response to oceanic warming, rather than a fundamental disagreement with observed trends.

### Upper-ocean salinity and stratification changes in the Southern Ocean

Since vertical transport of heat was found to be an important driver of upper-ocean warming, we now turn to the processes that control upper-ocean stratification. In the subpolar Southern Ocean, stratification is set primarily by salinity (*53*). We therefore begin by examining the salinity budget to identify the mechanisms governing changes in stratification.

**Fig. 5.**
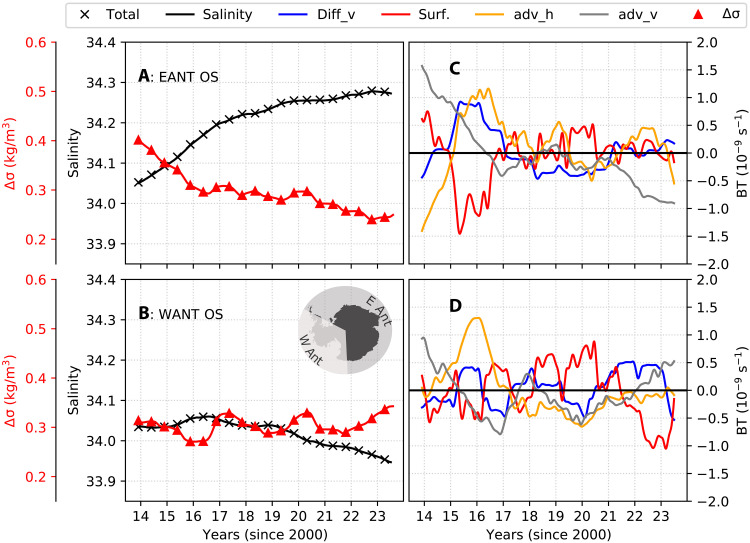
SOSE salinity budgets. (**A** and **B**) Salinity (black line with cross markers), vertically averaged over the upper 100 m and spatially averaged over the regions labeled within the panels. A 12-month rolling mean was applied to remove the seasonality. The time-integrated sum of the budget terms is shown by cross markers. Also shown are the stratification (quantified by $\sigma_{240}^{\theta }-\sigma_{0}^{\theta }$; red line with triangle markers), spatially averaged in each region. (**C** and **D**) Salinity budget terms (BT) are presented here as anomalies relative to their monthly means (see time-integrated budgets in fig. S9). Terms shown are the vertical advection (adv_v; gray lines), horizontal advection (adv_h; orange lines), vertical diffusion (Diff_v; blue lines), and surface fluxes (surf; red lines).

The upper ocean in East Antarctica displays a fresh anomaly from 2013 to 2015 (fig. S7). Thereafter, the regional upper ocean salinifies (Fig. 5A) and hosts an increase in the upper-ocean heat content. Part of the early freshening likely reflects enhanced equatorward export and melt of sea ice (*11*), with the subsequent salinification being consistent with a reduction in sea ice transport. In the following discussion, we show that upper-ocean salinification is initiated by the upward mixing of heat and salt, with an additional contribution arising from the later decrease in sea ice formation and the associated equatorward export and melt.

In West Antarctica, however, an anomalously saline upper ocean is initially present that progressively freshens from 2016 through 2023. As noted earlier, the loss in SIV in this region is less pronounced than in East Antarctica, with off-shelf areas showing a recovery in SIV after 2021. Such recovery is in accord with satellite-observed SIE anomaly maps, which indicate positive anomalies in parts of West Antarctica between 2016 and 2023 (fig. S14). The fresher upper ocean in West Antarctica is associated with positive anomalies in sea ice thickness during 2016, 2018, 2020, and from 2021 to 2023 (fig. S3). This is compatible with the SIV budget analysis, which showed a positive anomaly in the SIP term over the off-shelf West Antarctic during 2018–2021 (Fig. 2H).

### Salinity budgets off East Antarctica

We now examine spatially averaged salinities, and associated budget terms, to diagnose the causes of salinification over East Antarctica and of freshening over West Antarctica. East Antarctic near-surface salinification is associated with a decline in upper-ocean stratification (Δσ; defined as the difference between the potential density at depths of 240 and 0 m), with a minimum in 2023 (Fig. 5A). The increase in upper-ocean salinity is initially driven by the vertical advection term (gray line; Fig. 5C and fig. S8C), which exhibits a positive anomaly between 2013 and 2016. This term reflects the upwelling of salty waters and indicates a shoaling of the CDW layer, contributing to increased salinity in the uppermost 100 m. Vertical mixing also exhibits a positive anomaly in 2015 and 2016 (blue line; Fig. 5C) over off-shelf East Antarctica. Horizontal advection contributed to salinification over the off-shelf East Antarctic too during 2015–2016. Thus, both advective and diffusive terms played a role in the enhanced vertical and horizontal transfers of salt before 2016. We discuss the most likely driver of this salinification pathway in the following section.

Note that, while both vertical diffusion and vertical advection make important contributions to the salinity budget, in the heat budget, vertical diffusion dominates and vertical advection plays a more minor role. This difference arises because the vertical temperature gradient is roughly one to two orders of magnitude larger than the corresponding salinity gradient (fig. S10).

The surface flux term over off-shelf East Antarctica showed a positive anomaly during 2013–2014, followed by a negative anomaly in 2015–2016, and positive anomalies beyond 2016. These patterns correspond to anomalies in SIP over off-shelf East Antarctica (Fig. 2F), indicating that the surface flux anomalies were induced by variations in sea ice formation and transport. The positive anomalies in 2013–2014 reflect enhanced SIP, which increases salinity through brine rejection. In contrast, the negative anomalies in 2015–2016 coincide with reduced SIP, which led to upper-ocean freshening. Last, positive anomalies beyond 2016 correspond to reduced equatorward sea ice–derived freshwater transport (discussed in more detail in the “Synthesis” section). Surface fluxes and SIP are correlated at 0.68, with a 95% confidence interval of [0.64, 0.72] (numbers in square braces throughout the manuscript denote the 95% confidence interval of correlation after correcting for autocorrelation and removing seasonality and trend). The upper-ocean freshening resulted from both diminished brine rejection and increased export of sea ice from the continental shelf of East Antarctica, which melts during summer and deposits freshwater over the off-shelf regions.

### Salinity budgets off West Antarctica

Salinity in the upper ocean over West Antarctica does not exhibit the increasing tendency seen over East Antarctica. Instead, salinity over the continental shelf of West Antarctica peaks in 2015 and 2016 and freshens thereafter, with an associated enhancement in stratification. The freshening and increased stratification are more pronounced over the continental shelf of West Antarctica, with the off-shelf West Antarctic experiencing a slight freshening in the years 2019–2023, with an associated enhancement of stratification.

West Antarctic freshening is explained by the persistent negative anomaly in the vertical advection term through the years 2015–2021 over the off-shelf West Antarctic (Fig. 5D) and during 2017–2019 over the West Antarctic continental shelf (fig. S8D). Starting in 2020 and continuing through 2023, the vertical advection term shows signs of strengthening over both the continental shelf and off-shelf regions of West Antarctica.

### Surface stress forcing of salinity and stratification changes

To assess the drivers of the upper-ocean salinity and stratification changes shaping sea ice evolution, we revisit the vertical and horizontal advection terms of the salinity budget and compare them with surface stresses in SOSE. Surface stresses on the ocean reflect the combined effects of winds and sea ice drift. Vertical salinity advection is expected to respond to Ekman pumping driven by the cyclonicity of surface stresses, while meridional salinity advection in the upper ocean is anticipated to respond to zonal stresses (*54*). This analysis is restricted to the off-shelf regions of East Antarctica and West Antarctica. When computing the stress curl, the full (zonal and meridional) surface stress vector was considered, whereas anomalies in zonal stresses were computed using only positive (eastward) values. This approach is equivalent to using a spatial mask that selects the area with westerlies, which drive eastward stress and northward Ekman advection—a mechanism that has been proposed as a driver of sea ice loss (*18*).

**Fig. 6.**
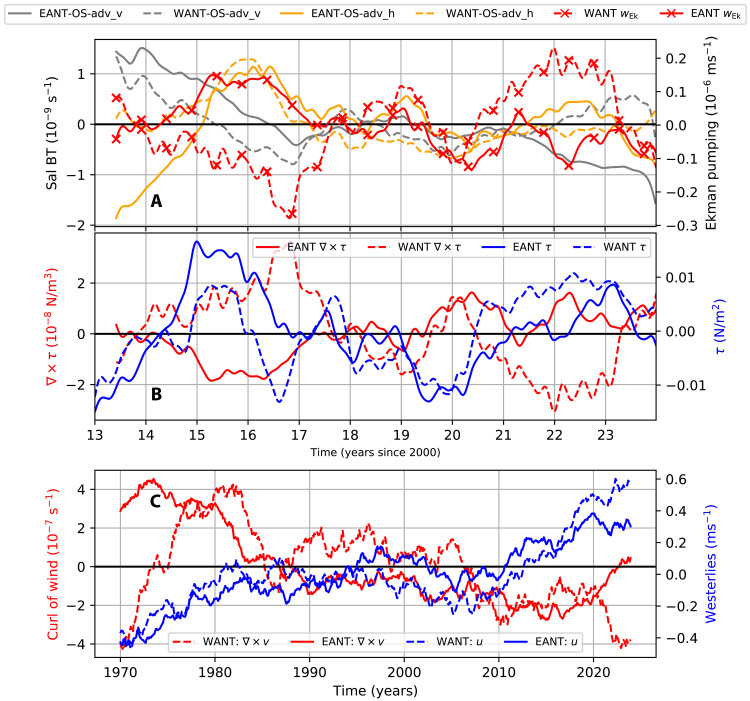
Surface stresses and advection. (**A**) Vertical and horizontal salinity advection terms [reproduced from Fig. 5 (E to H)], and the Ekman pumping velocity (*w*_Ek_), presented as anomalies for off-shelf regions in E Ant and W Ant. (**B**) Anomalies in the surface stress and stress curl over off-shelf regions in E Ant and W Ant. (**C**) Anomalies in the curl of ERA5 wind velocity and in the ERA5 westerlies over the off-shelf regions of E Ant and W Ant. Time series in (A) and (B) are smoothed with a 12-month rolling mean to highlight interannual variability, while (C) uses a 10-year rolling mean to emphasize decadal trends.

As expected, during periods of enhanced cyclonicity in surface stress (negative anomalies in ∇ × τ; Fig. 6B), the vertical salinity advection term exhibits anomalously positive values (gray lines in Fig. 6A). Conversely, during periods of weakened cyclonicity in surface stress (positive anomalies in ∇ × τ; Fig. 6B), the vertical advection term shows negative anomalies. SOSE reveals a zonal asymmetry in the stress curl: East Antarctica experiences intensified cyclonicity from 2013 to 2017, followed by a weakening; whereas West Antarctica exhibits weak cyclonicity from 2014 to 2017, which intensifies thereafter. This zonal asymmetry in the surface stress curl corresponds to a similar asymmetry in vertical advection across East and West Antarctica from mid-2021 to 2023. In East Antarctica, surface stress curl and vertical advection of salinity are correlated at −0.26 [−0.33, −0.19], while in West Antarctica, they are correlated at −0.28 [−0.35, −0.21]. We note here that although Ekman pumping and vertical salinity advection are related through the large-scale circulation, their tendencies need not always align (Fig. 6). Ekman pumping reflects the vertical velocity induced by the curl of the surface wind stress, whereas vertical salinity advection depends on both this vertical velocity and the local vertical salinity gradient.

A zonal asymmetry is also found in the intensity of the eastward stress over East Antarctica and West Antarctica. Both regions exhibit an intensification between 2014 and 2016 and again from 2020 to 2023 (Fig. 6B). However, the zonal stresses are generally stronger off East Antarctica between 2014 and 2016, whereas during 2020–2023, they are regularly more intense off West Antarctica. The horizontal advection terms in East and West Antarctica covary with the temporal variations of the eastward stress, suggesting that Ekman advection plays a role in driving horizontal salinity advection anomalies in the upper ocean. In East Antarctica, the horizontal advection of salinity and eastward zonal stresses are relatively strongly correlated at 0.62 [0.57, 0.67], while in West Antarctica, they are relatively weakly correlated at 0.12 [0.05, 0.19]. Once again, we note that Ekman advection does not fully explain the horizontal salinity advection. Other processes, such as changes to the wind-driven gyre variability (*55*) and changes to the overturning circulation (*31*), could also contribute.

To contextualize these results within the multidecadal changes in Southern Ocean climate, we analyze ERA5 wind fields (monthly averages at 10 m above sea level) from 1970 to 2023 over the off-shelf regions of East and West Antarctica (Fig. 6C). Only the eastward component of the zonal wind is considered in computing “WANT: u” and “EANT: u.” Wind curl and westerlies were calculated as anomalies relative to their monthly means and smoothed using a 10-year rolling mean to capture interdecadal variability. The subpolar Southern Ocean has experienced a long-term intensification of wind curl over both East and West Antarctica. In addition, the westerlies have shown a sustained positive trend, reaching their highest magnitudes during the period from 2010 to 2023. The long-term trends in the winds indicate an intensification in the processes that induce upward transport of heat and salt from the subsurface ocean.

### Synthesis

The changes in Antarctic SIE, and in upper-ocean hydrography and stratification, documented in the preceding sections may be synthesized into three distinct periods: P1 (mid-2013 to 2014), P2 (2015–2017), and P3 (2018–2023). These periods were selected on the basis of the temporal evolution of SIV in the off-shelf East Antarctic (Fig. 2B), as this is the main contributor to total Antarctic SIV changes. P1 corresponds to a period of elevated SIV. P2 marks the onset of SIV loss. P3 captures the persistence of a low SIV state with further decline.

**Fig. 7.**
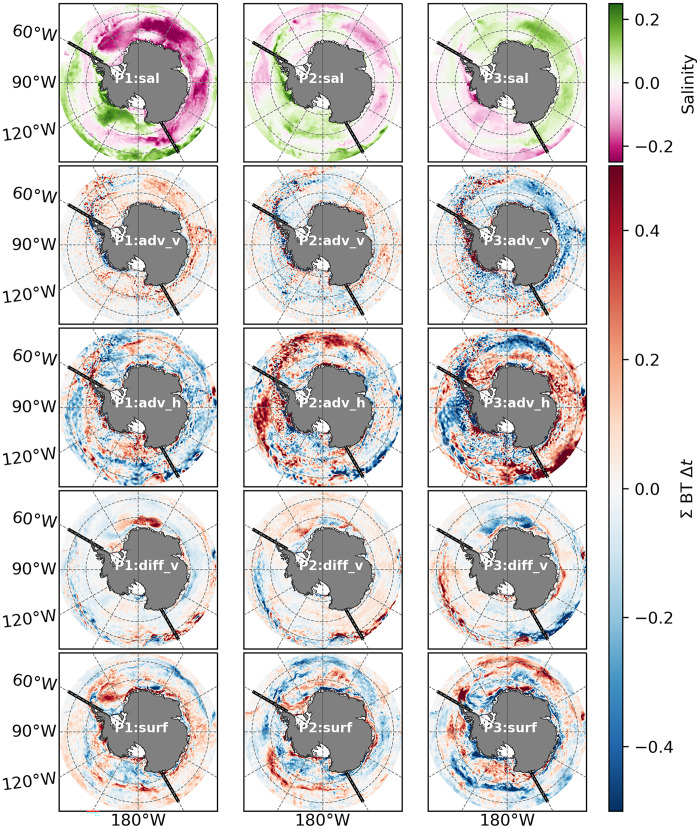
Salinity budget maps. Time-integrated over periods: P1 (01 June 2013 through 2014), P2 (2015–2017), and P3 (2018–2023). Top row shows salinity anomalies, and subsequent rows show the time-integrated salinity budget terms for vertical advection (adv_v), horizontal advection (adv_h), vertical diffusion (diff_v), and surface fluxes (surf). All terms are on the unit-less practical salinity scale (*81*). Black thick lines mark the boundaries between E and W Ant at longitudes 150°E and 60°W.

During P1, the upper ocean exhibits a fresh anomaly across much of East Antarctica, while the West Antarctic region displays a saline anomaly (Fig. 7; P1-sal). In P2, the magnitude of these anomalies diminishes overall. P3 is characterized by a salty anomaly in East Antarctica and a fresh anomaly in West Antarctica. Saline and fresh anomalies are respectively associated with reduced and strengthened upper-ocean stratification.

During P1 (before 2015), the only budget term that displays a positive (salinifying) anomaly over the entire subpolar Southern Ocean is the vertical advection term (Fig. 7; P1-adv_v). The surface flux term exhibits a positive anomaly everywhere except in the Ross Sea, off-shelf regions of the Amundsen and Bellingshausen Seas, and the Eastern Weddell Sea. A positive surface flux anomaly is consistent with enhanced brine rejection due to stronger SIP in this period.

P2 (2015–2017) is characterized by sea ice loss, accompanied by an upper-ocean salinity increase across much of East Antarctica. The vertical advection term continues to salinify the upper ocean over large areas of East Antarctica, consistent with increased cyclonicity in the surface stress curl during this period. Surface fluxes show a freshening tendency near the sea ice margins, consistent with increased sea ice import from the continental shelf followed by melting offshore. Surface fluxes also show a salinifying tendency within the ice pack in East Antarctica. In general, Ekman advection (which is a component of horizontal advection) moves cooler and fresher waters northward from high-latitude regions. However, during P2, horizontal advection partly counteracts the surface fluxes by enhancing salinity along the northern margins of the off-shelf regions while exhibiting a freshening tendency in areas further south. This pattern occurs alongside positive anomalies in eastward surface stresses in East Antarctica and a strong negative anomaly in surface stresses in West Antarctica during 2016. P2 is also characterized by increased vertical diffusion (Fig. 7; P2-diff_v) across much of the subpolar Southern Ocean, associated with intensified surface stresses that likely promote vertical mixing (see EANT-τ in Fig. 6B).

P3 exhibits a reversal of the zonal asymmetry in upper-ocean salinity, with salty anomalies in East Antarctica and fresh anomalies in West Antarctica (Fig. 7; P3-sal). Vertical advection shows a freshening tendency, consistent with reduced cyclonicity in the surface stress curl over much of East Antarctica. In contrast, over the off-shelf West Antarctic, vertical advection exhibits a slight positive anomaly during this period. Salinification due to horizontal advection strengthens, aligning with enhanced surface stresses and increased northward Ekman advection of salt over much of East Antarctica, partly counteracting the vertical advection-induced freshening. Surface fluxes indicate a salinifying tendency across the bulk of East Antarctica, consistent with decreased SIP in both the off-shelf and continental shelf regions. This leads to reduced sea ice (and freshwater) import into the off-shelf areas. In parts of the Ross and Amundsen Seas in West Antarctica, however, surface fluxes freshen along the pack margins and salinify within the sea ice field, consistent with increased SIP on the continental shelves and reduced production and more melting in the off-shelf regions.

It is notable that our diagnosed mechanism during P3, wherein the surface fluxes act to salinify the upper ocean due to reduced sea ice export into the off-shelf East Antarctic regions, is the reverse of that reported by Haumann *et al.* (*11*) during 1982–2008. At that time, sea ice export was enhanced, leading to a freshening of the off-shelf regions. This reversal is in line with the oppositely signed tendencies in SIE in 1982–2008 versus P3.

## DISCUSSION

We have shown that Antarctic sea ice loss in recent years was the compound result of a range of drivers acting in three distinct phases (Fig. 7). This has led to a sustained low sea ice state unprecedented in the observational record (i.e., since the 1970s). These phases were most clearly observed in East Antarctica. At the start of the first phase (P1; 2013–2014), the upper ocean in East Antarctica was relatively cool and fresh (Fig. 7; P1:sal). This was qualitatively consistent with the immediate response described by the two-timescale hypothesis (*39*), although the hypothesis was based on idealized simulations that do not entirely capture the full complexity of the freshwater cycle that SOSE reveals. Further, P1 is also in accord with the Haumann *et al.* (*11*) mechanism, wherein an expansion in SIE caused freshening on the margins of East Antarctica. However, during P1 and P2, vertical advection and mixing progressively increased the upper ocean’s salinity and heat content, leading to a saltier and warmer state from P2 (2015–2017) through P3 (2018–2023) that qualitatively aligns with the longer-timescale response anticipated by the two-timescale hypothesis. Thus, our analysis suggests that the subsurface ocean played an important role in initiating upper-ocean warming and salinification that ultimately led to a pronounced sea ice loss in East Antarctica.

In addition, we identify a third stage (P3) characterized by the response of surface fluxes to sea ice loss (Fig. 7; P3:surf). This response results in salinification of off-shelf regions due to a reduced import of freshwater via sea ice—a process analogous to a reversal of the Haumann *et al.* (*11*) mechanism.

Sea ice loss in East Antarctica was initiated via heat input through the shoaling and mixing of heat from the CDW layer below the pycnocline (Fig. 3). From 2015 to 2020, sea ice loss over the off-shelf regions resulted in increased SIP and freshwater export from the continental shelves. However, after 2020, SIP declined in both the off-shelf and continental shelf regions, leading to salinification due to reduced sea ice and freshwater import into the off-shelf areas of East Antarctica. These salty anomalies were partly redistributed by Ekman advection, forced by anomalous eastward surface stresses associated with intensified westerlies. Once sea ice was lost, the albedo feedback mechanism amplified heat gain through enhanced absorption of shortwave radiation. Doddridge *et al.* (*16*) used numerical experiments to show that this excess heat penetrates into the subsurface during summer and is reentrained into the mixed layer during the subsequent winter, inhibiting sea ice growth.

**Fig. 8.**
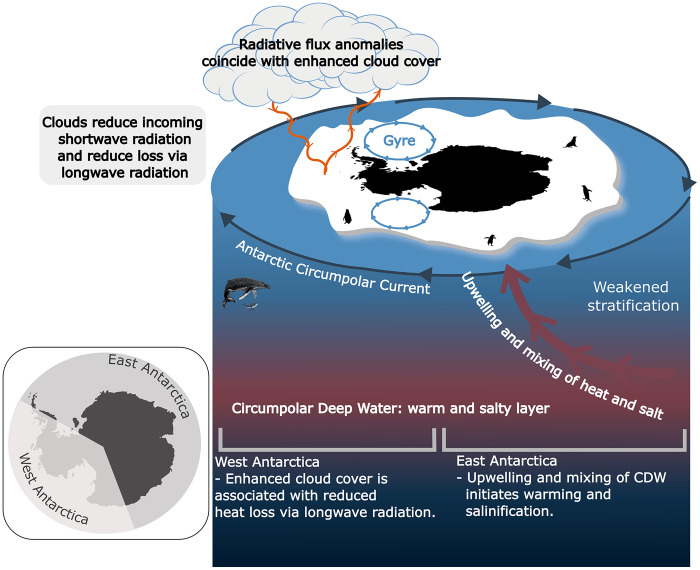
Summary of processes driving Antarctic sea ice loss. In West Antarctica, radiative flux anomalies associated with enhanced cloud cover in years 2016, 2017, 2019, and 2020 coincided with the inception of sea ice loss. In East Antarctica, shoaling of the warm and salty CDW and the subsequent mixing of heat into the mixed layer during years 2013–2016 initiated sea ice loss and eroded upper-ocean stratification.

Using heat budgets, we showed that upper-ocean warming in East Antarctica was primarily initiated by the mixing of heat from below the pycnocline, whereas in West Antarctica, it was induced by enhanced downward longwave radiation associated with increased cloud cover. Schroeter *et al.* (*33*) demonstrated that the intensification of the ZW3 pattern enhanced the meridional transport of warm subtropical air masses toward the Ross, Amundsen, and Bellingshausen Seas from 2007 to 2021. Such meridional transport has been shown to affect sea ice via enhanced longwave radiative fluxes (*33*, *56*). Further, Josey *et al.* (*12*) found that sea ice loss during 2023 was concentrated in regions of strong meridional transport and was associated with enhanced ocean-to-atmosphere heat loss. A summary of processes identified in this study is provided in Fig. 8.

The different balance of processes in East versus West Antarctica is manifested in a zonal asymmetry in upper-ocean salinification, which was driven by contrasting patterns of wind forcing between the two regions. A similar zonal asymmetry has been reported by an observational study spanning the period 2005 to 2025, which found that the CDW layer shoaled in the Weddell Sea and East Antarctica, while a deepening of the CDW layer took place over West Antarctica (*57*). In our analysis, West Antarctica begins with a saltier upper ocean, possibly due to enhanced Ekman pumping consistent with more intense wind curl over this region from 2009 to 2012 (Fig. 6C). West Antarctica then gradually freshens after 2016, partly influenced by negative anomalies in the vertical advection term, which align with weaker surface stress curl over the region through much of the period from 2014 to 2021. In contrast, East Antarctica experiences an enhancement in the cyclonicity of surface stresses, leading to increased Ekman pumping of salty warm waters toward the surface.

West Antarctica also experienced low sea ice conditions associated with upper-ocean saline anomalies. However, SIV loss was less pronounced than in East Antarctica and showed a recovery in the off-shelf regions of West Antarctica after 2021. Observed SIE also displayed a recovery in 2020 and 2021 but declined again thereafter. The West Antarctic upper ocean salinified only weakly in 2022 and 2023, although the vertical advection term supplied salt at a greater rate during this period (P3; 2018–2023; Fig. 7). This salinification was driven by enhanced cyclonicity in the surface stress curl (Fig. 6B). The slow response of upper-ocean salinity to this vertical advection may explain the apparent discrepancy in SOSE’s SIE during these years.

The SOSE run period captures the sea ice–ocean dynamics over the most recent decade, which encompasses the abrupt reduction in Antarctic sea ice in 2016. However, upwelling-favorable conditions occurred over a much longer period: a multidecadal trend toward enhanced cyclonicity in the winds over the subpolar Southern Ocean began in the 1980s (Fig. 6C). Such intensification in wind cyclonicity coincided with a period of positive SAM that strengthened the surface stresses (*24*). The Southern Ocean has also seen a multidecadal shoaling of the CDW layer and an accumulation of heat in the subsurface (*58*–*60*).

Enhanced wind stress and cyclonicity were found to precede anomalously low sea ice conditions in a model-based study (*42*). Reconstructions of past sea ice suggest a reduction in SIE in the 1970s that took place under similar conditions of enhanced SAM and cyclonicity, with a greater loss seen over East Antarctica relative to West Antarctica (*61*).

A substantial caveat to consider is that the regions that we define as West and East Antarctica contain notable internal variability, as illustrated in Fig. 7. For example, West Antarctica spans a marked zonal asymmetry: The Amundsen and Bellingshausen Seas lie beneath the poleward branch of atmospheric flow associated with the ASL, whereas the Ross Sea is influenced by equatorward air flow. Sea ice in these regions shows opposing links to the ASL (*62*). While our analysis focuses on large-scale, circumpolar dynamics, a more detailed subregional investigation will be necessary to fully resolve aspects of localized variability in the sea ice–ocean–atmosphere system around Antarctica. In addition, we have not explicitly considered other important processes—such as anthropogenic warming (*63*), eddy heat fluxes (*64*), and changes in Southern Ocean storminess (*20*)—all of which have been shown to influence sea ice.

Is the current decline in Antarctic sea ice a signal of a new regime in Southern Ocean dynamics, potentially locking the system into a persistent low sea ice state? Statistical analyses show evidence of a regime evolution in sea ice from 2007 onward, finding increased variance and autocorrelation (*65*) and increased persistence in summer minima from year to year (*46*). Sea ice vorticity coupling with wind vorticity has increased, possibly indicating thinner sea ice overall (*66*). Predicting the future evolution of Southern Ocean sea ice requires caution, as climate models often struggle to accurately represent the complex processes governing the lifecycle of sea ice and its interplay with Southern Ocean dynamics, largely due to their coarse grid resolution and crude mixing parameterization schemes (*67*).

Nevertheless, there is good reason to believe that upwelling-favorable conditions, driven by an enhanced SAM and intensified cyclonicity, are likely to persist under the influence of greenhouse gas emissions and the ozone hole (*28*, *39*). These conditions are expected to facilitate upward mixing of CDW heat into the upper ocean, reinforcing the present low sea ice state. At the same time, under anthropogenic forcing, the ocean is expected to freshen due to enhanced precipitation (*35*, *36*) and glacial freshwater runoff (*68*). The future evolution of Antarctic sea ice will depend on the balance between wind-driven upwelling and ocean destratification on the one hand and freshwater-driven surface restratification on the other.

## METHODS

### Southern Ocean state estimate

The Biogeochemical SOSE is based on the MITgcm numerical ocean model with data assimilation through the adjoint method (*69*). We used iteration-155 for this analysis (accessed from https://sose.ucsd.edu/), which has a horizontal resolution of 1/6° and 52 unevenly spaced vertical levels and which runs from 2013 to 2023. The model iteratively assimilates in situ hydrographic profiles from Argo and tagged seals and remotely sensed sea surface height, sea surface temperature, and sea ice concentration. The assimilation does not introduce any unphysical nudging terms and is carried out via the adjustment of the model’s boundary forcing and initial conditions, hence preserving the budgets of conservative quantities. The atmospheric parameters were prescribed from ERA5 fields (*70*) at hourly intervals using boundary layer bulk formulae (*71*). The sea ice model represents the viscous-plastic rheology of ice and the thermodynamic equations governing its growth (*72*). Continental meltwater runoff is prescribed from the dataset in Hammond and Jones (2016) (*73*), which is a multiyear average of freshwater fluxes from Antarctic ice shelves and ice sheets. This approach captures regional variation in meltwater discharge but does not account for its temporal variability, which can be substantial in some regions (*74*). This limitation is mitigated, to some extent, by the fact that our discussion is focused on off-shelf regions and basin-scale processes, where the effects of winds, Ekman advection, and vertical mixing dominate the heat and salt budgets.

SOSE hydrography (*45*), sea ice properties (*75*), and thermodynamics (*3*) have been validated by numerous studies. Here, we extend the validation by comparing SOSE sea ice characteristics with satellite-based observations. Wintertime sea ice in SOSE is displaced equatorward compared to satellite data and shows a negative bias in the Weddell Sea, particularly pronounced between 60°W and 60°E (fig. S13). However, when examining the anomalies of each product relative to their own 11-year monthly-mean, the locations of positive and negative sea ice concentration anomalies are comparable between the two datasets (fig. S14). Although the magnitude of the anomalies in SOSE is larger, possibly affecting the trend, the SIE anomalies summed over the entire Southern Ocean are comparable across the two datasets (fig. S14, bottom). This provides confidence in our use of the state estimate to explore the mechanisms behind the observed sea ice loss. We note here that the sea ice biases may emerge from the model’s upper-ocean turbulence parameterization scheme (*76*) and from biases within the atmospheric product used (ERA5) (*77*), although SOSE has been shown to reduce these systematic biases through the assimilation scheme (*78*).

We further compare SOSE hydrography with objectively mapped fields of observations (EN4.2.2) (*79*) in the Supplementary Materials (figs. S11 and S12). Owing to the scarcity of observations in the Southern Ocean, this comparison is necessarily limited. Moreover, EN4.2.2 is itself subject to uncertainties arising from the smoothing inherent in its objective mapping scheme and the tendency for the solution to relax toward a climatological background in poorly sampled regions such as the Southern Ocean (*79*). Where observations are available (observational weight *w* > 0.9), SOSE generally appears biased toward a fresher (fig. S11) and cooler (fig. S12) upper ocean. These biases do not aid the mechanisms of sea ice decline discussed in the manuscript, as they are opposite in sign to the upper-ocean warming and salinification associated with the decline. However, in regions with lower observational weights (0.5 < *w* < 0.9; dotted regions in figs. S11 and S12) such as the Weddell Sea (between longitudes 30°W and 60°E), SOSE appears biased toward a warmer and saltier upper ocean, possibly explaining the negative bias in sea ice concentrations seen in this region. Yamazaki *et al.* (*80*) have noted that SOSE exhibits a warm bias at around 100-m depth, but we use vertical averages of the upper 100 m of the water column to minimize the impact of this warm bias.

We restrict this analysis to the model domain south of 50°S. The model is fully equilibrated in terms of kinetic energy convergence, but the first 6 months of model output during 2013 are discarded, as the model hydrography and sea ice require time to evolve from the influence of the initial conditions. The analysis is spatially averaged over two regions: (i) West Antarctica (W Ant), defined between longitudes 150°E and 300°E, encompassing the Ross, Amundsen, and Bellingshausen Seas; and (ii) East Antarctica (E Ant), which includes all regions outside of W Ant, covering the Weddell Sea and East Antarctica. In addition, we define continental shelf regions as areas shallower than 3000 m and south of 60°S, with off-shelf regions comprising all areas beyond these criteria. We use a spatial mask that selects only regions that lie within the 11-year averaged September SIE.

We consider the salinity (equivalent to salt concentration) budget

$$\frac{\text{∂}S}{\text{∂}t}=G_{\text{adv}\_v}+G_{\text{adv}\_h}+G_{\text{diff}\_h}+G_{\text{diff}\_v}+G_{\text{surf}}$$ (1)

where the terms on the right-hand side represent the salinity tendency due to vertical advection ($G_{\text{adv}\_v}$), horizontal advection ($G_{\text{adv}\_h}$), horizontal diffusion ($G_{\text{diff}\_h}$), vertical diffusion ($G_{\text{diff}\_v}$), and net surface fluxes ($G_{\text{surf}}$) due to evaporation, precipitation, meltwater runoff, and sea ice. All terms were stored online as 5-day averages. All salinity values are on the unit-less practical salinity scale (*81*), and the budget terms are in units of $s^{-1}$ (per second).

Similarly, a temperature (equivalent to heat) budget is evaluated

$$\frac{\text{∂}\theta }{\text{∂}t}=G_{\text{adv}\_v}+G_{\text{adv}\_h}+G_{\text{diff}\_h}+G_{\text{diff}\_v}+G_{\text{surf}}$$ (2)

where the time tendency of potential temperature (θ in degrees Celsius) is diagnosed as a function of vertical advection ($G_{\text{adv}\_v}$), horizontal advection ($G_{\text{adv}\_h}$), horizontal diffusion ($G_{\text{diff}\_h}$), vertical diffusion ($G_{\text{diff}\_v}$), and net surface fluxes ($G_{\text{surf}}$) of heat. The net surface fluxes integrate all surface heat flux components, i.e., sensible, latent, longwave, and shortwave radiative fluxes. In this analysis, we separate out the shortwave component from the remaining surface components (surf). The budget terms are in units of degrees Celsius per second. As for the salinity budget, all temperature budget terms were stored online as 5-day averages.

The SIV budget is computed as (*49*)

$$\frac{\text{∂SIV}}{\text{∂}t}=-\text{AD}+\text{SIP}$$ (3)

using the advection [**u**_**i**_ ·∇(SIV)] and divergence [SIV (∇·**u**_**i**_)] of sea ice, where **u**_**i**_ is the horizontal sea ice velocity. The residual was used to represent the in situ SIP. The sum of AD of SIV represents the mechanical movement of sea ice, while SIP represents the thermodynamic growth and melt of sea ice.

The Ekman pumping velocity is calculated using the total surface stress: $w_{\text{Ek}}=\frac{1}{\rho_{0}}\nabla \times \left(\frac{\tau }{f}\right)$, where *w*_Ek_ is the vertical Ekman pumping velocity, the density of seawater ρ_0_ = 1035 kg m^−3^, τ is the surface horizontal stress on the ocean from wind and sea ice, and f=2Ω sin(ϕ) is the Coriolis parameter, with the planetary angular velocity being $\Omega =2\frac{\pi }{T}$, and the planet’s rotational period as *T* = 86,400 s.

### Other methodological considerations

Satellite-observed sea ice concentration was obtained at daily frequency from the National Snow and Ice Data Center (*82*), and wind data were acquired as monthly averages from the ERA5 reanalysis (*70*). Throughout this manuscript, all budget terms were smoothed using a 12-month rolling mean and a Butterworth filter with a window size of 90 days before being plotted. This was done to remove the seasonal signal, as the focus of this study is on interannual and longer-timescale variability. However, correlations were computed on the unfiltered time series to avoid autocorrelations introduced through rolling averages. Numerical values represented as anomalies were computed within each spatial grid cell against the 11-year (2013–2023) monthly mean, after which they were spatially averaged. The budget terms presented as anomalies do not sum to the absolute value of the scalar. Instead, the cross markers on the time series indicate the cumulative sum of the budget terms. We provide a more detailed explanation of this methodological choice in the Supplementary Materials.
