# Supplementary material for: Compound drivers of Antarctic sea ice loss and Southern Ocean destratification
Source: Sci Adv. 2026 May 8;12(19):eaeb0166. doi: 10.1126/sciadv.aeb0166 (PMC13155358; doi:10.1126/sciadv.aeb0166)
Supplement: Supplementary file 1 — Supplementary Text Figs. S1 to S15 [file sciadv.aeb0166_sm.pdf]

Science Advances
AAAS

Supplementary Materials for

Compound drivers of Antarctic sea ice loss and Southern Ocean destratification

Aditya Narayanan *et al.*

Corresponding author: Aditya Narayanan, a.narayanan@soton.ac.uk

Sci. Adv. **12**, eaeb0166 (2026)
DOI: 10.1126/sciadv.aeb0166

This PDF file includes:

Supplementary Text
Figs. S1 to S15

Seasonal Variability in the Subpolar Southern Ocean

We provide the unfiltered potential temperature (Figure S15A) and salinity (Figure S15B) budgets. Here, the seasonal cycle is the most prevalent feature, involving the growth and melt of sea-ice – which corresponds to salinification (due to brine rejection during the formation of sea-ice in autumn) and freshening (due to the melting of sea-ice in spring). The peak in upper-ocean salinity and a temperature minimum are reached in September (when the annual maximum sea-ice extent is attained), and the upper ocean is freshest and warmest in February (when sea-ice extent reaches its annual minimum).

The dominant terms in the salinity budget are the surface fluxes, balanced by vertical mixing (Diff_v). Similarly, the dominant terms in the heat budget are the surface flux and vertical mixing terms, which once again largely offset each other. However, although the other budget terms are smaller in magnitude, they cannot be neglected, as they also contribute to interannual variability due to the large cancellation between the dominant terms. This is the reason why we present the anomalies in the budget terms in the main text and in Figures S6 and S9, making their relative contributions to the overall salt and heat tendencies more obvious.

SOSE Hydrography Compared with EN4 climatology

We provide comparisons of SOSE hydrography with objectively mapped fields from the Hadley Centre's EN4.2.2 product (hereafter EN4) in Figures S11 and S12. The EN4 product relaxes the temperature and salinity fields to climatology when observations are unavailable. Hence, to allow comparison with robust observations, we provide hatching over regions with observational weights below 0.5, and dots over regions with observational weights between 0.5 and 0.9. Observation weights range from 0 to 1, denoting the weight provided to observations versus the background climatology (*79*). Regions with no markers indicate high observational weights, but they may still suffer from interpolation errors. In regions with sufficiently high observational weights (> 0.9), the upper ocean (vertical averages over uppermost 100 m) appears to be fresher and cooler in SOSE. The cold bias ranges from -0.21ºC to -0.05ºC, and the fresh bias ranges from -0.21 to -0.06.

Additional notes on the warming and salinification of the continental shelf

The continental shelves of East Antarctica also exhibit a warming tendency, although this is slower and less pronounced than in the off-shelf regions (Figure S5A). The warming is initially driven by the vertical mixing term (Diff_v; Figure S5C). Surface fluxes (surf) align with changes in sea-ice volume in this region (Figure 2C), exhibiting a warming tendency during phases of greater sea-ice volume and a cooling tendency during periods of reduced sea-ice volume. Shortwave fluxes display a prominent positive anomaly from 2021 to 2023, coinciding with substantial sea-ice volume loss in these regions. Once again, a warming tendency via shortwave fluxes lags the initial onset of sea-ice loss. The continental shelves of West Antarctica exhibit patterns similar to those seen in East Antarctica, where reduced sea-ice volumes correspond to reduced surface heat fluxes and increased shortwave influx, most prominently seen in 2017, 2022 and 2023 (Figure S5D).

The continental shelf of West Antarctica experienced a positive anomaly in the salinity vertical advection term from 2020 to 2023, but this was balanced by a negative anomaly in the horizontal advection term (Figure S8D). As a result, there is no change in upper-ocean salinity across these years except toward the end of 2023, when salinity increased and stratification decreased.

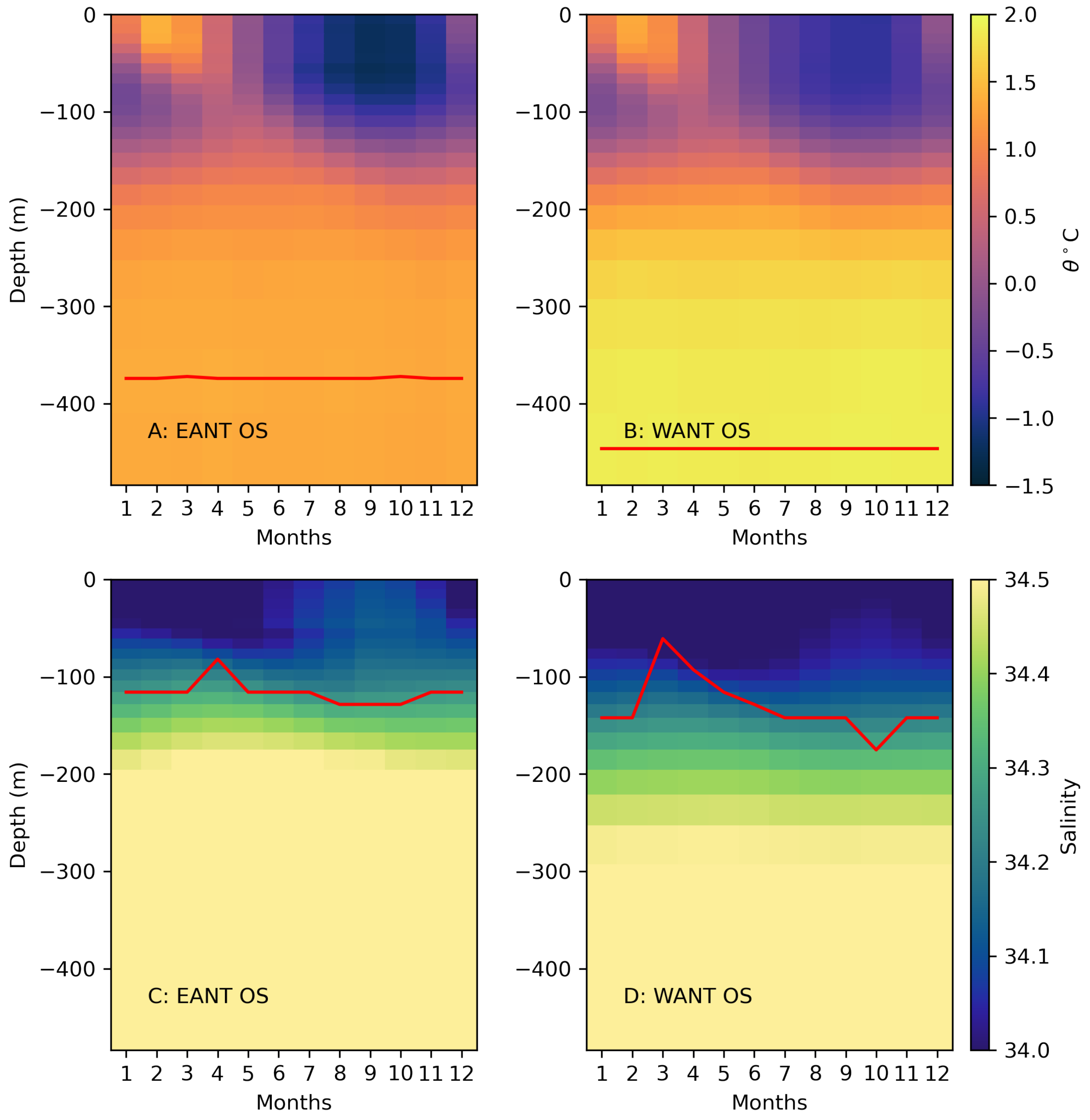

Figure S1: **EN4 climatological monthly average** (A-B) potential temperature and (C-D) salinity in the offshore East and West Antarctica. Red contours in temperature represent the temperature maximum layer, while the red contours in the salinity panels represent the internal pycnocline depth.

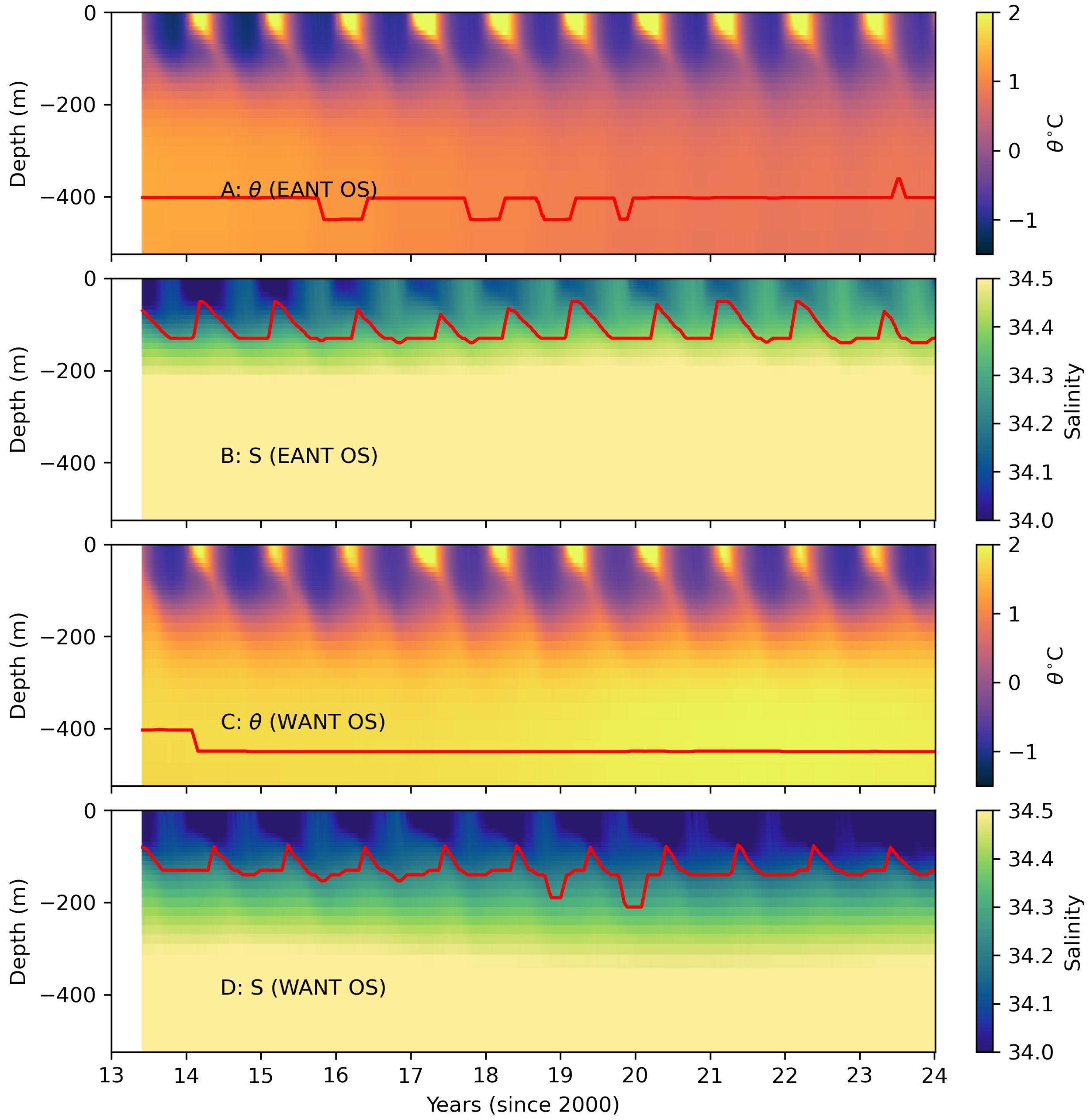

Figure S2: **SOSE hydrography.** SOSE potential temperature and salinity in (**A-B**) East Antarctica's off-shelf (EANT OS) and (**C-D**) West Antarctica's off-shelf (W Ant OS) regions. Red contours in the temperature panels represent the temperature maximum layer, while the red contours in the salinity panels represent the internal pycnocline depth.

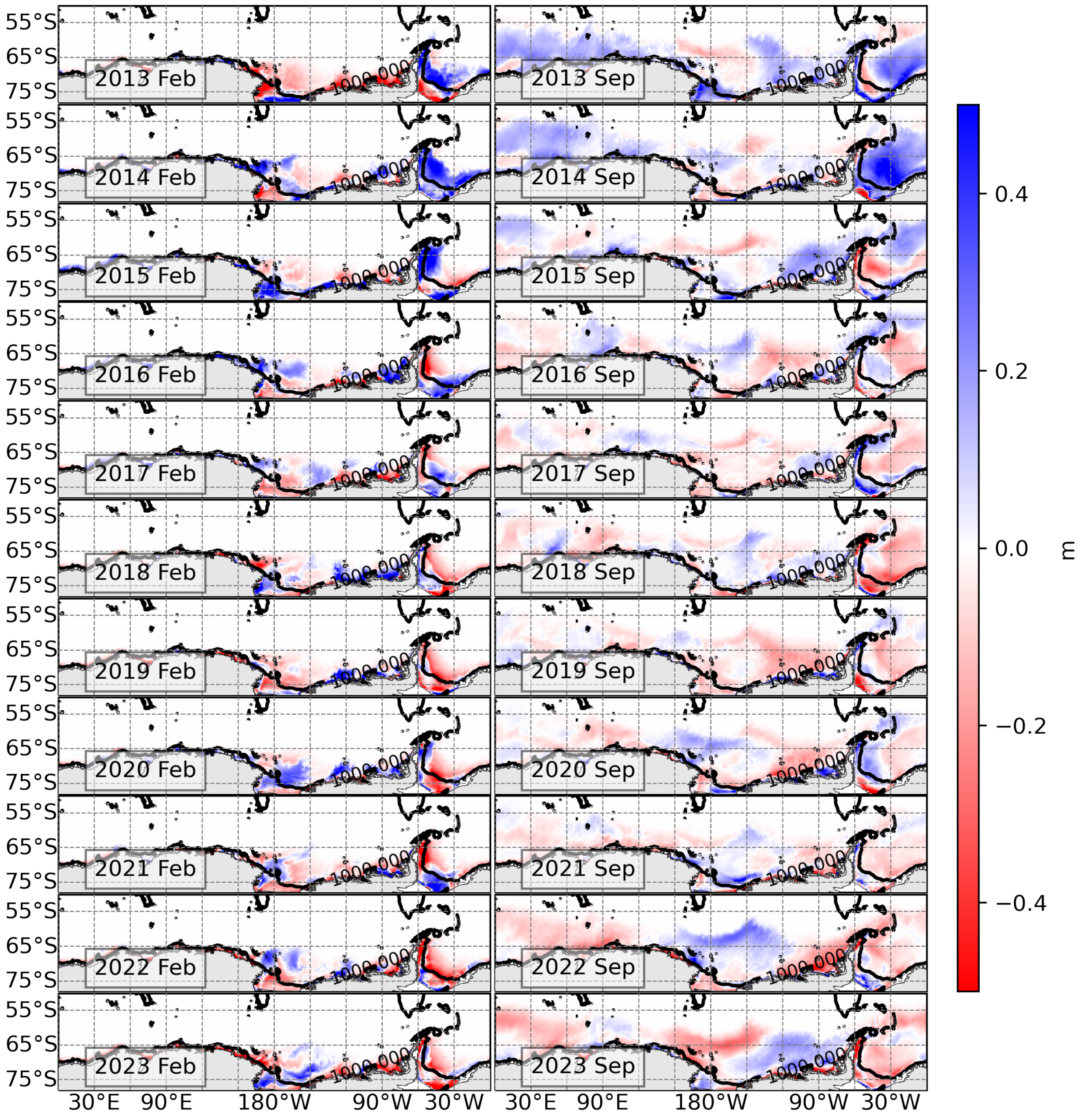

Figure S3: **Sea-ice thickness from SOSE** represented as anomalies with respect to the 11-year monthly means, shown here for February (left hand column) and for September (right hand column).

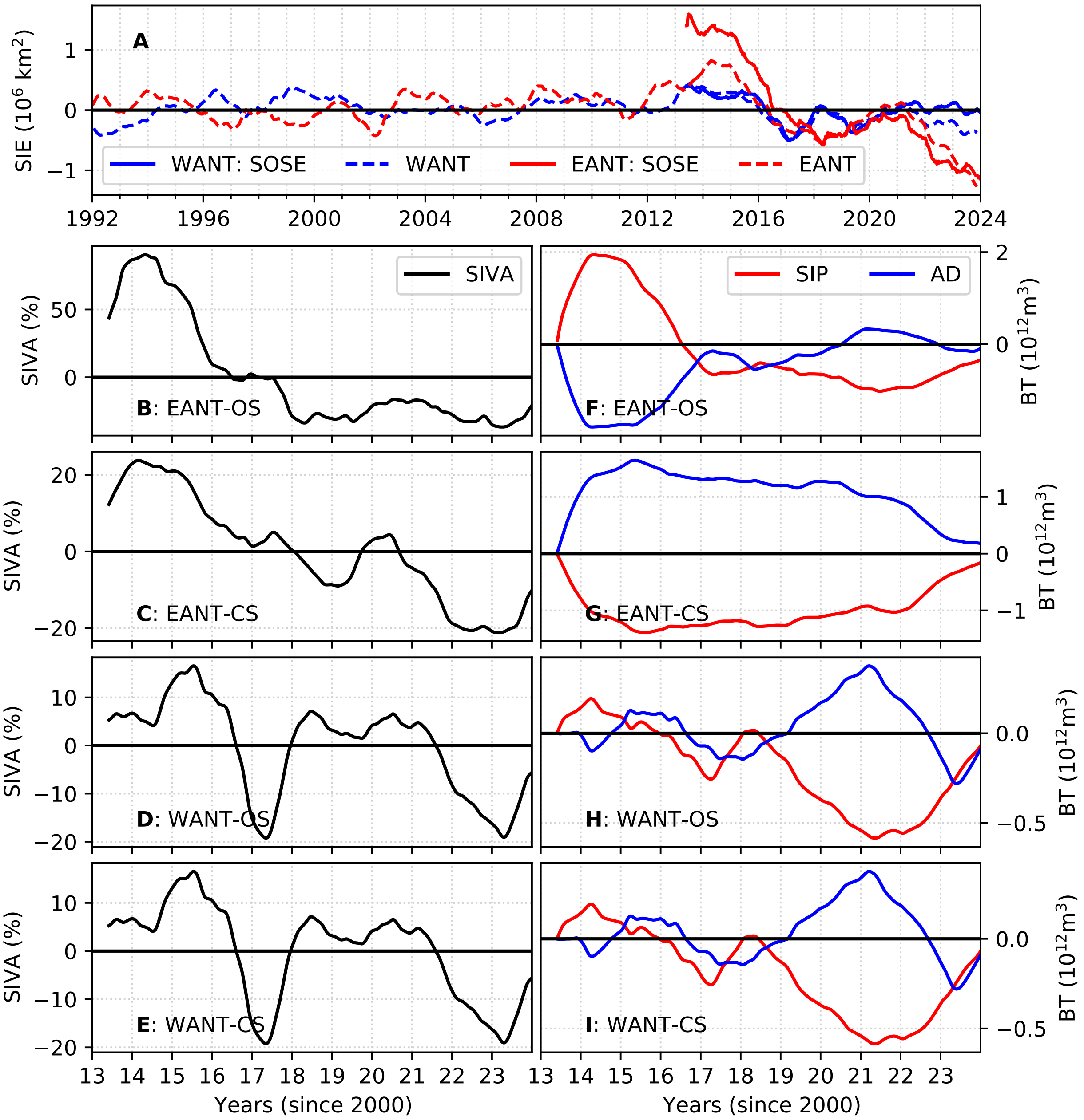

Figure S4: **Sea-ice budgets.** (**A**) Sea-ice extent anomalies computed from satellite observations (broken line) and SOSE (solid lines) for E Ant (EANT; red lines) and W Ant (WANT; blue lines). (**B-E.**) Sea-ice volume anomalies (represented as percentage fraction of the monthly-mean volume), spatially summed over the continental shelf (CS) and off-shelf (OS) regions of East Antarctica and West Antarctica. (**F-I.**) Time-integrated sea-ice volume budget terms (BT) for the corresponding regions are shown on the right hand column. The terms, represented as anomalies against the 11-year monthly mean, are the sea-ice production (SIP; red line; computed as a residual of Equation 2) and the advection and divergence (AD; blue line) of sea-ice volume.

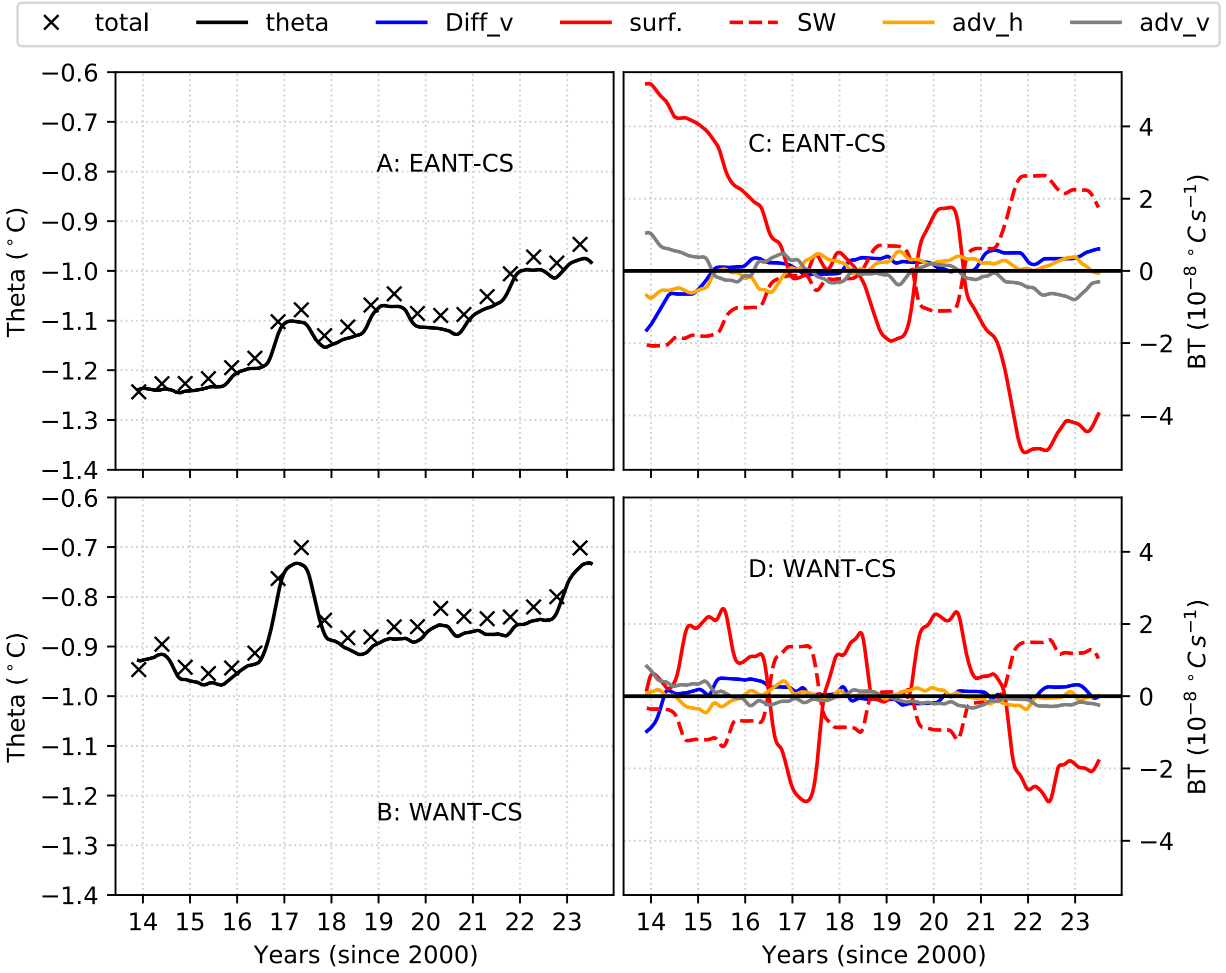

Figure S5: **SOSE temperature budgets on the continental shelf.** (**A-B.**) Potential temperature (θ; black line with cross markers), vertically averaged in the upper 100 m of the water column, and spatially averaged over the regions labeled within the panels. A 12-month rolling mean was applied to remove the seasonality. The time-integrated sum of the budget terms is shown by cross markers. (**C-D.**) θ budget terms are presented here as anomalies relative to their monthly means. Terms shown are the vertical advection (adv_v; grey lines), horizontal advection (adv_h; orange lines), vertical diffusion (Diff_v; blue lines), surface fluxes (surf; red lines), and shortwave fluxes (SW; broken red lines).

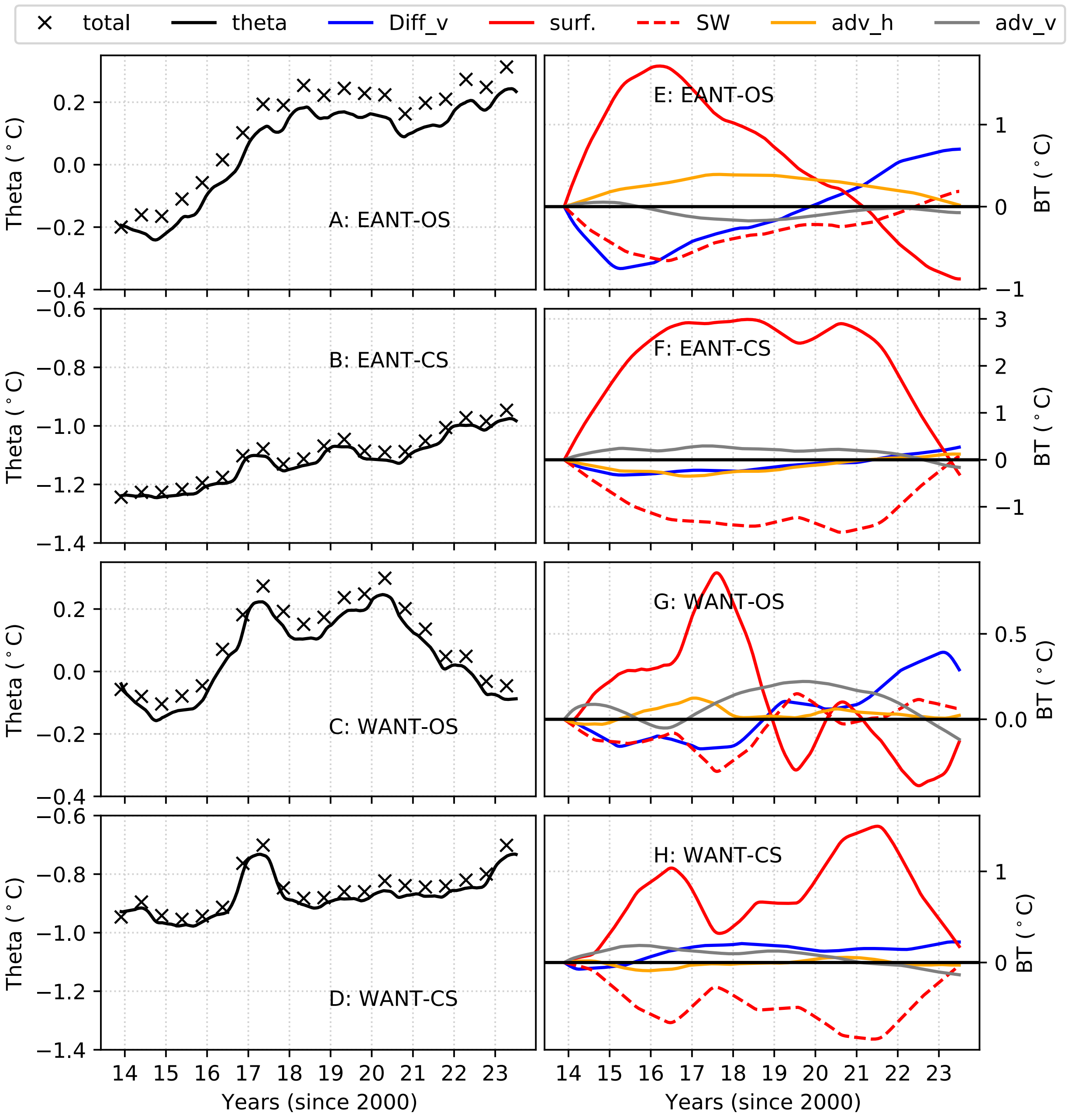

Figure S6: **SOSE time-integrated temperature budgets.** (**A-D.**) Potential temperature (θ; black line with cross markers), vertically averaged in the upper 100 m of the water column, and spatially averaged over the regions labeled within the panels. A 12-month rolling mean was applied to remove the seasonality. The time-integrated sum of the budget terms is shown by cross markers. (**E-H.**) θ budget terms are computed as anomalies relative to their 11-year monthly means and then integrated in time. Terms shown are the vertical advection (adv_v; grey lines), horizontal advection (adv_h; orange lines), vertical diffusion (Diff_v; blue lines), surface fluxes (surf; red lines), and shortwave fluxes (SW; broken red lines).

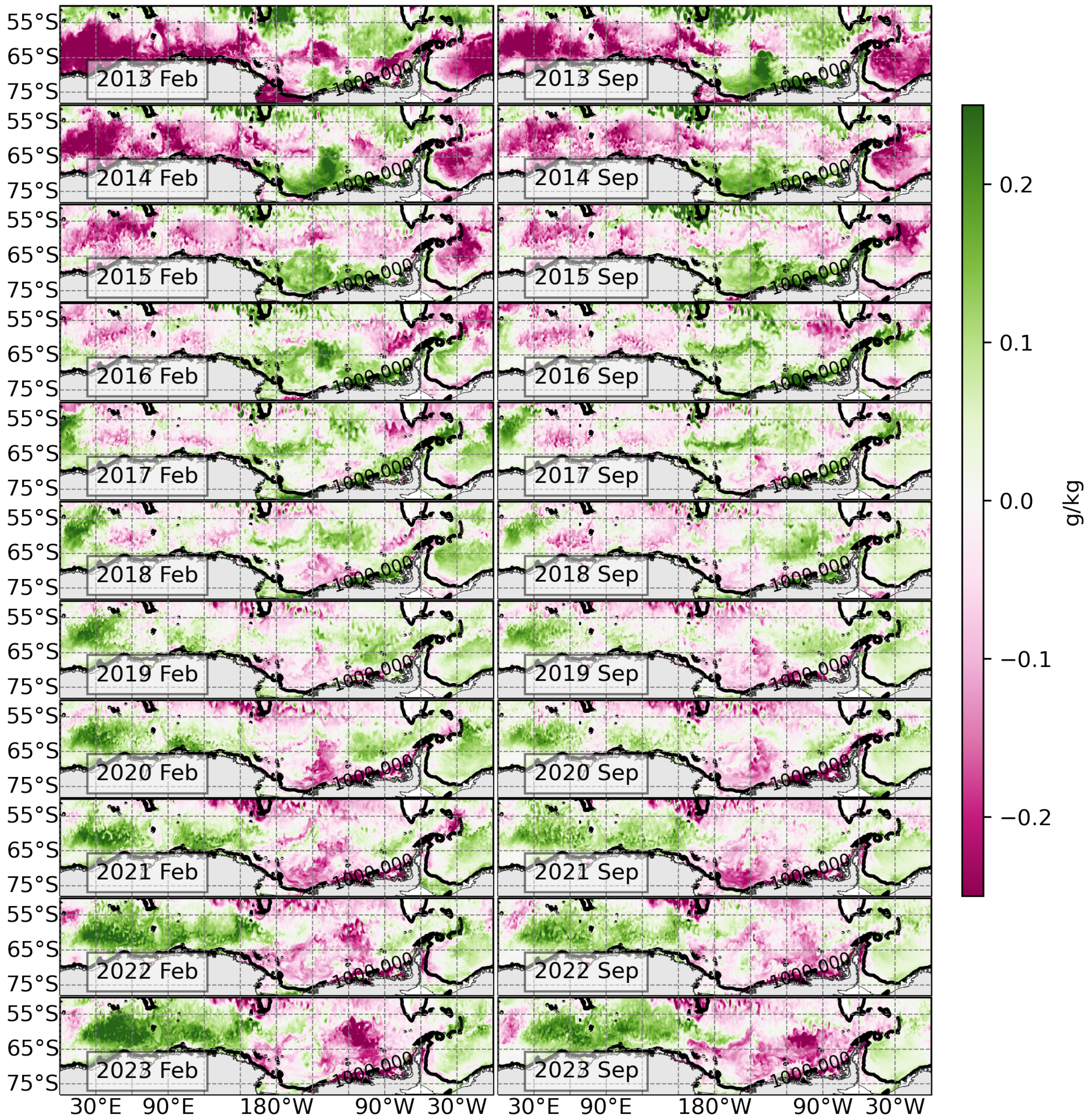

Figure S7: **Upper-ocean (0-100 m) salinity anomaly** during February (left hand column) and September (right hand column).

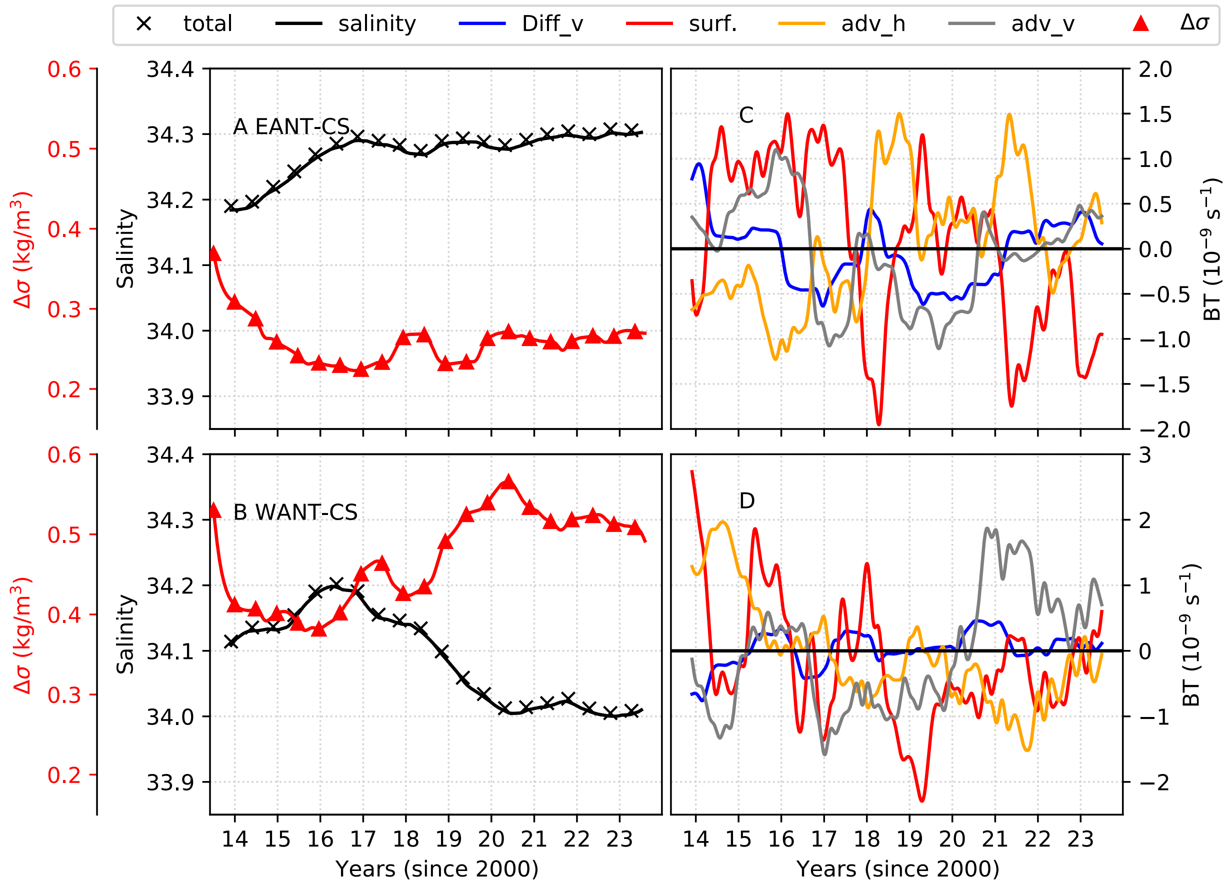

Figure S8: **SOSE salinity budgets on the continental shelf:** (**A-B.**) Salinity (black line with cross markers), vertically averaged over the upper 100 m, and spatially averaged over the regions labeled within the panels. A 12-month rolling mean was applied to remove the seasonality. The time-integrated sum of the budget terms is shown by cross markers. Also shown are the stratification (quantified by $\sigma^{\theta}_{240} - \sigma^{\theta}_{0}$; red line with triangle markers), spatially averaged in each region. (**C-D.**) Salinity budget terms are presented here as anomalies relative to their monthly means (see time-integrated budgets in Figure S9). Terms shown are the vertical advection (adv_v; grey lines), horizontal advection (adv_h; orange lines), vertical diffusion (Diff_v; blue lines), and surface fluxes (surf; red lines).

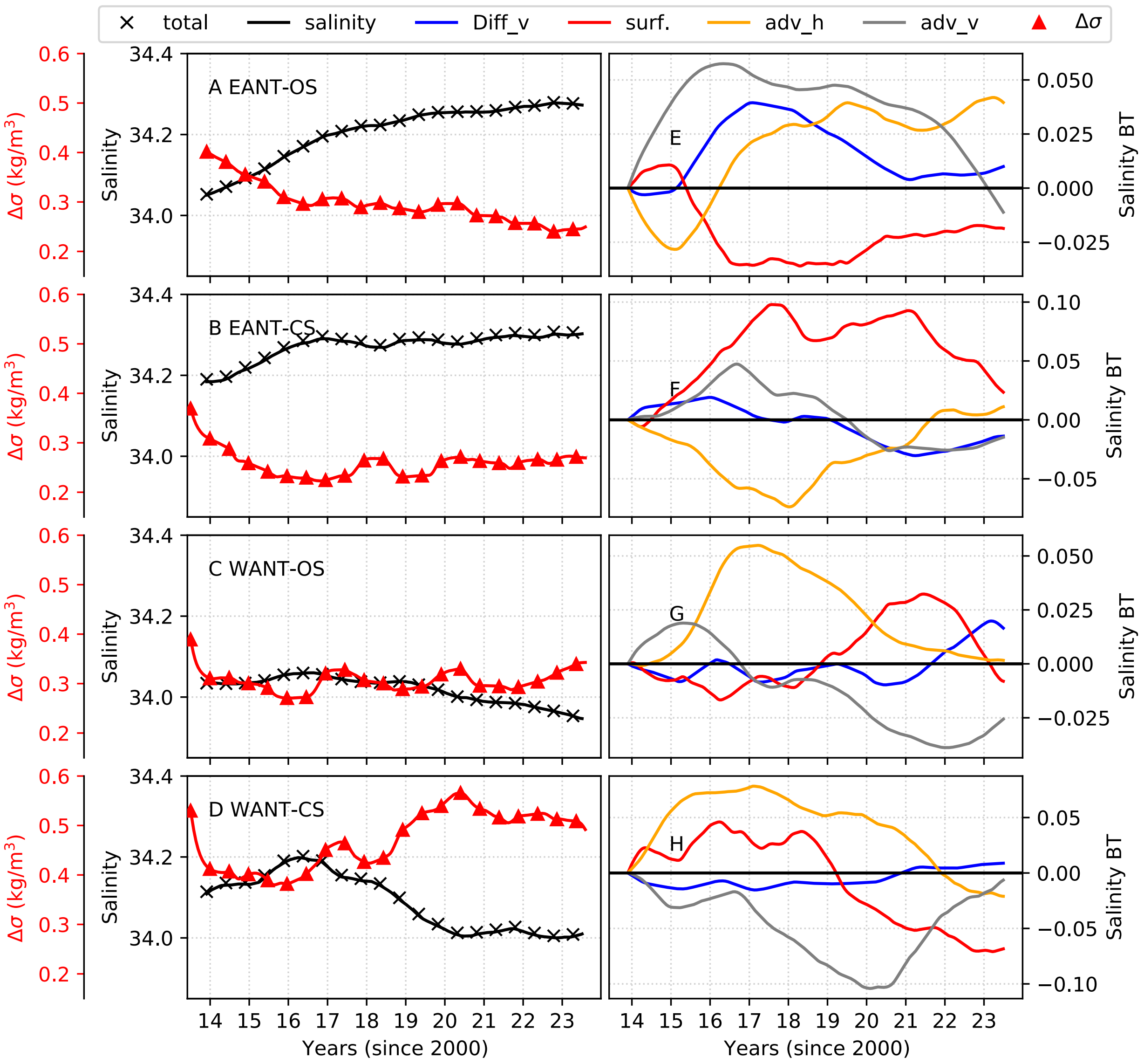

Figure S9: **SOSE time-integrated salinity budgets.** (**A-D.**) Salinity (black line with cross markers), vertically averaged over the upper 100 m, and spatially averaged over the regions labeled within the panels. A 12-month rolling mean was applied to remove the seasonality. The time-integrated sum of the budget terms is shown by cross markers. Also shown are the stratification (quantified by $\sigma_{240}^{\theta} - \sigma_{0}^{\theta}$; red line with triangle markers), spatially averaged in each region. (**E-H.**) Salinity budget terms (BT) are computed as anomalies relative to their 11-year monthly means and then integrated in time. Terms shown are the vertical advection (adv_v; grey lines), horizontal advection (adv_h; orange lines), vertical diffusion (Diff_v; blue lines), and surface fluxes (surf; red lines).

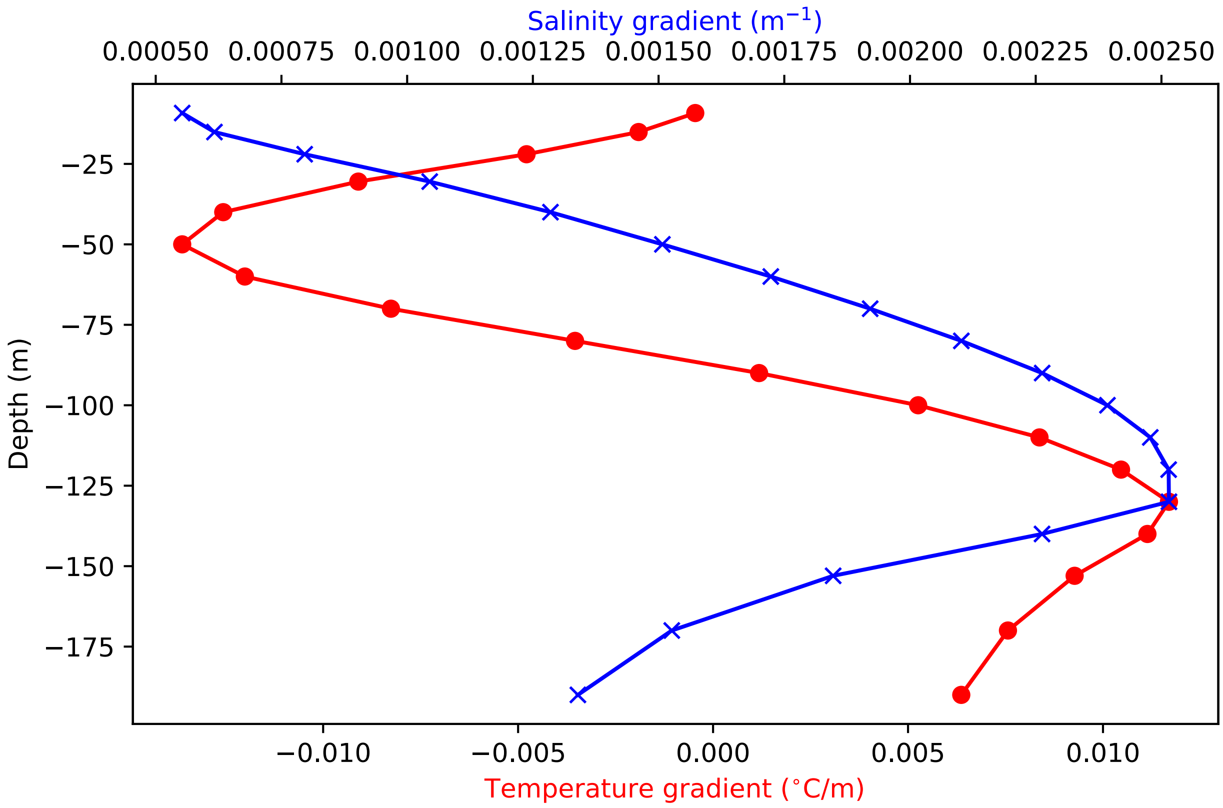

Figure S10: **Climatological vertical gradient in SOSE** of temperature (shown in red with circular markers; read bottom X axis) and salinity (shown in blue with cross markers; read top X axis), spatially averaged in E Ant (offshelf regions).

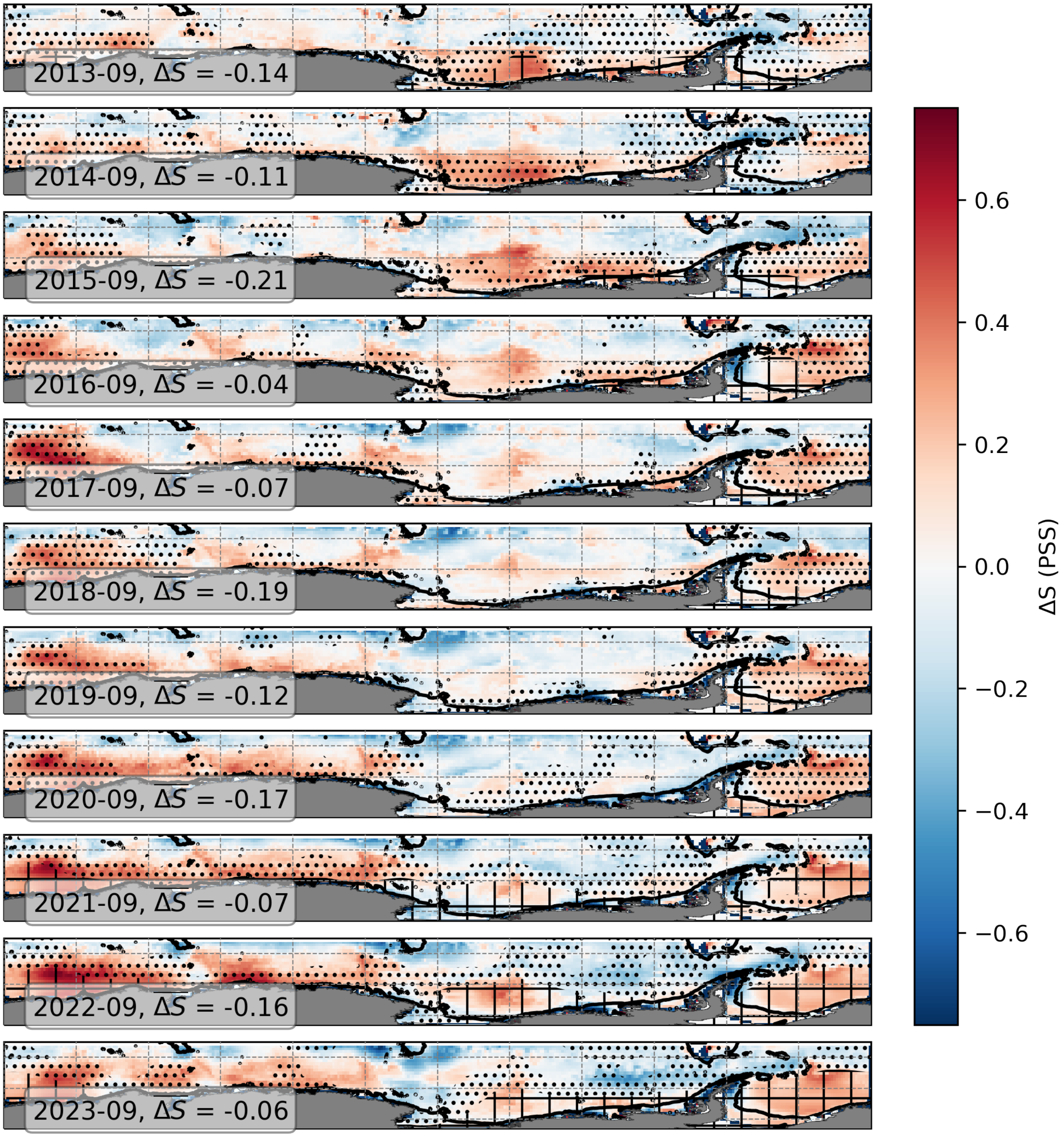

Figure S11: **SOSE minus EN4 salinity** averaged over the uppermost 100 m during September of each year. Regions with observational weights below 0.5 are hatched, while regions with weights between 0.5 and 0.9 are marked with dots. Clear regions have high weights.

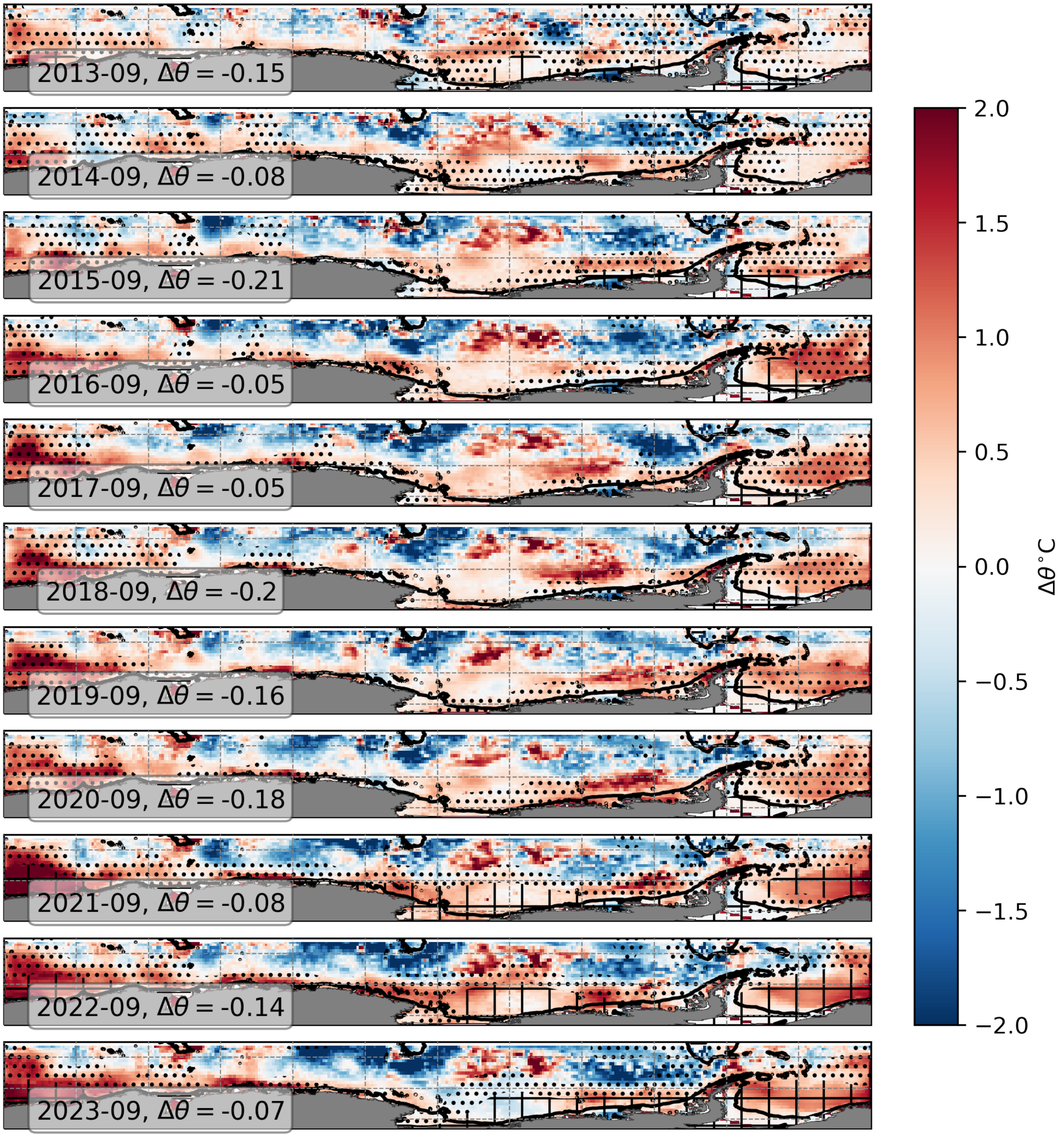

Figure S12: **SOSE minus EN4 potential temperature** averaged over the uppermost 100 m during September of each year. Regions with observational weights below 0.5 are hatched, while regions with weights between 0.5 and 0.9 are marked with dots. Clear regions have high weights.

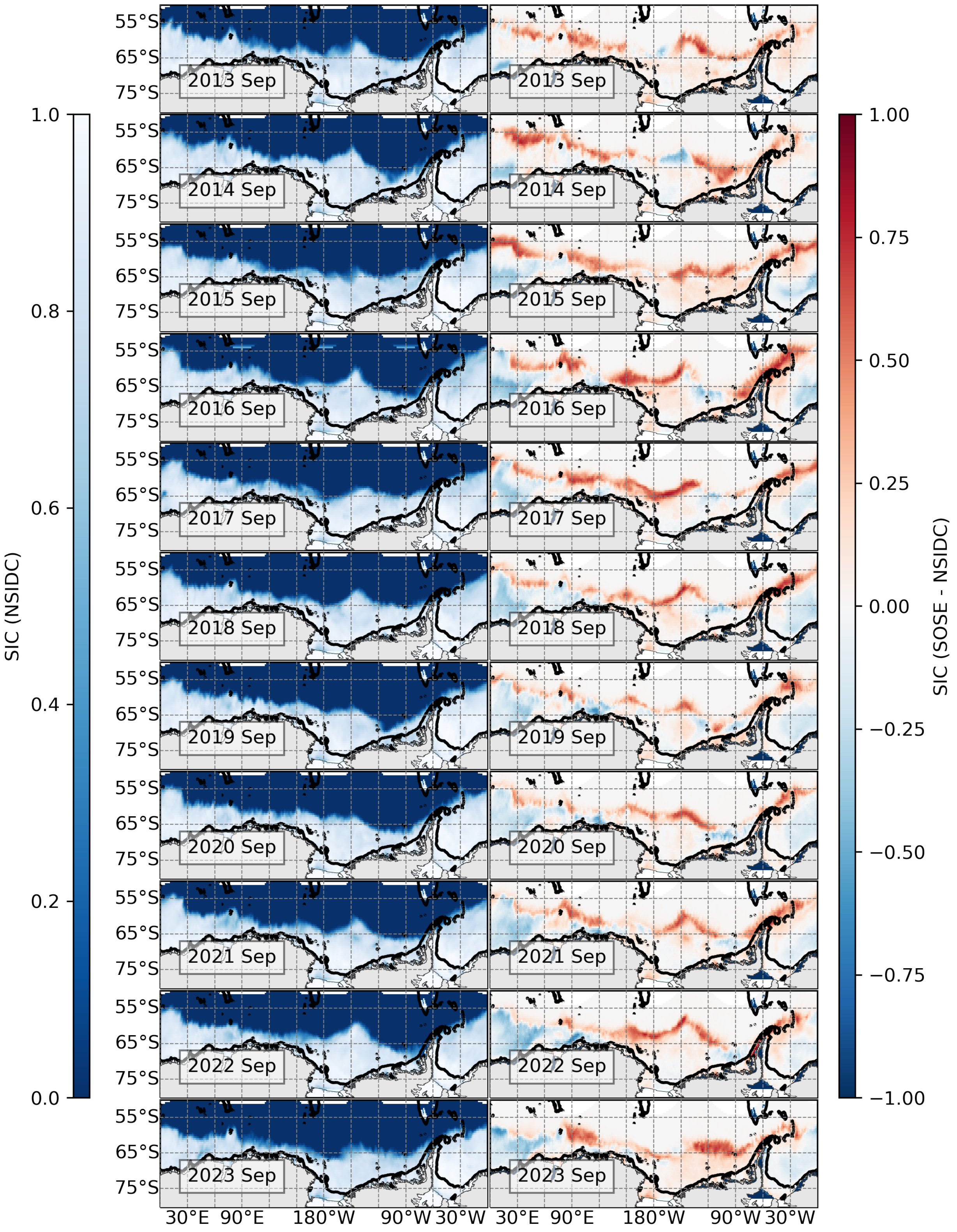

Figure S13: **Sea-ice concentration (SIC) measured by satellites**, averaged in September, is shown on the left hand column. SOSE SIC minus satellite-observed SIC, averaged in September, is shown on the right hand column.

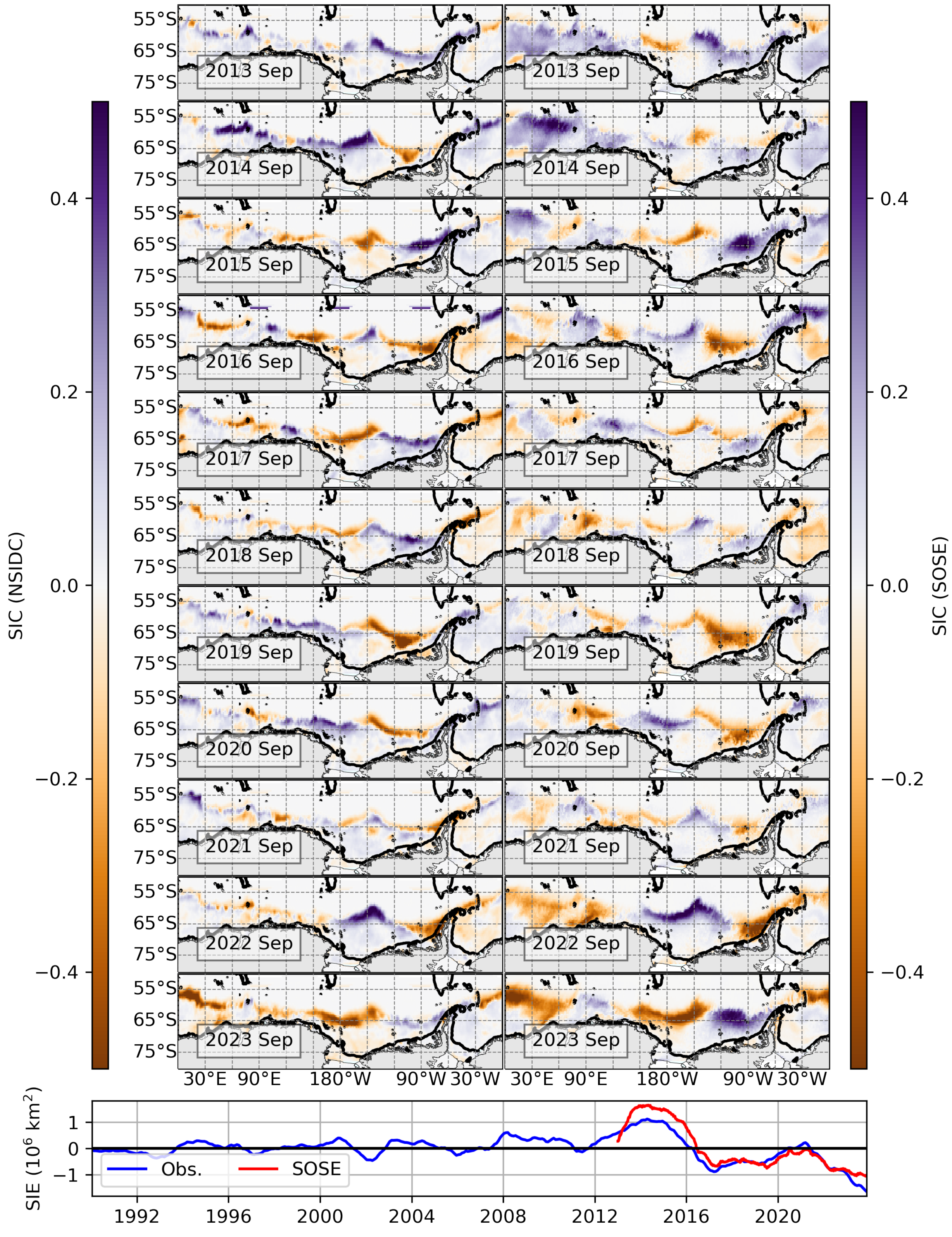

Figure S14: Sea-ice concentration anomaly relative to the September mean (2013-2023) for satellite observations (left column) and for SOSE (right column). Lower panel shows the sea-ice extent (SIE) anomaly in satellite observations (Obs.; blue line) and in SOSE (red line).

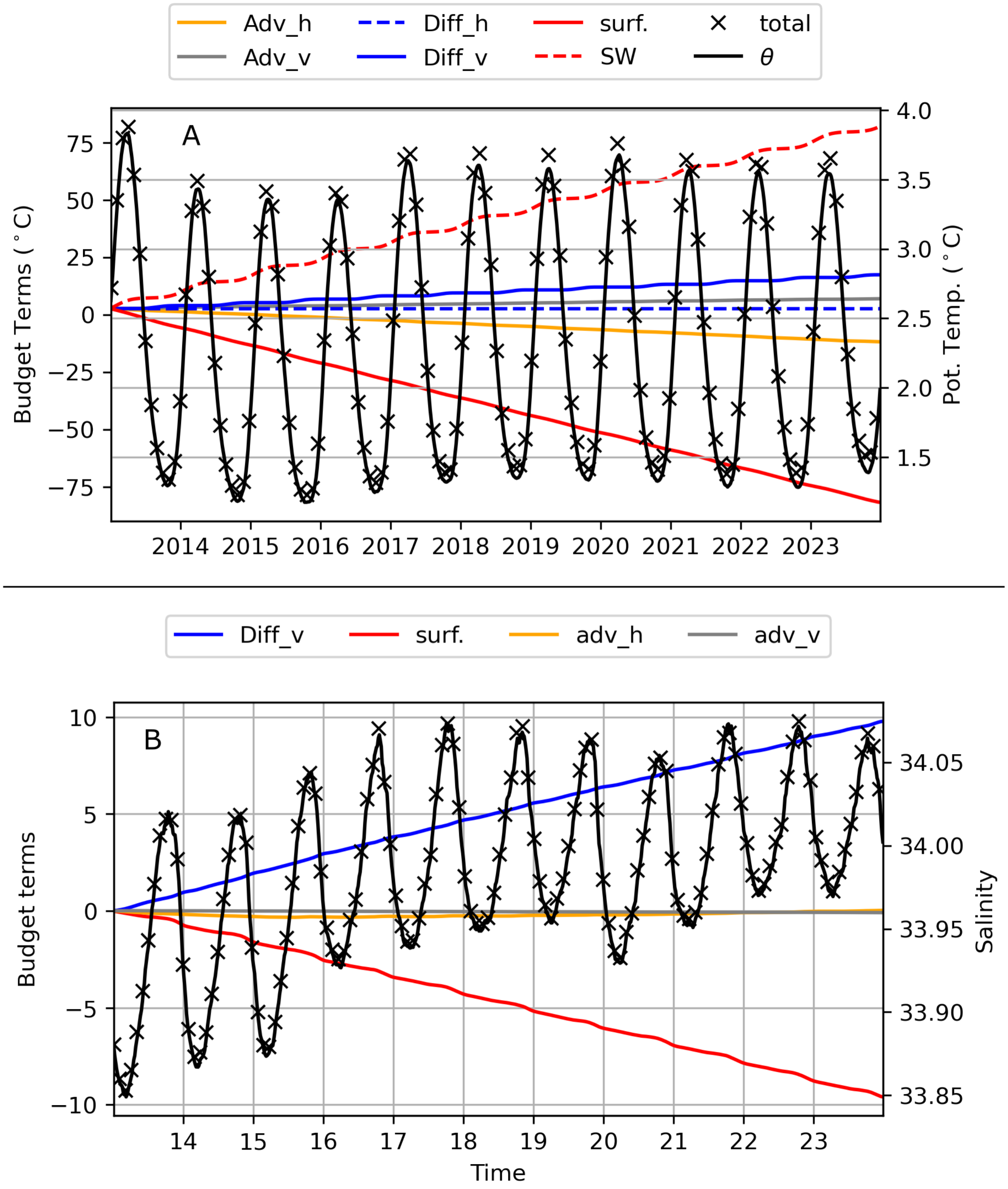

Figure S15: **Seasonal variability in temperature and salinity budgets** (averaged between 50ºS and 65ºS). **A** Potential temperature and its budget; and **B** salinity and its budget. Both plots are vertically averaged over 0-100 m with time-integrated budget terms.
